# RNA-seq analysis reveals the genes/pathways responsible for genetic plasticity of rice to varying environmental conditions on direct-sowing and transplanting

**DOI:** 10.1038/s41598-022-06009-w

**Published:** 2022-02-10

**Authors:** Suresh Kumar, Karishma Seem, Santosh Kumar, Trilochan Mohapatra

**Affiliations:** 1 Division of Biochemistry, ICAR-Indian Agricultural Research Institute, New Delhi, 110012 India; 2 Decode Genomics Private Limited, New Delhi, India; 3 Indian Council of Agricultural Research, New Delhi, India

**Keywords:** Biochemistry, Plant sciences

## Abstract

Rice cultivation by transplanting requires plenty of water. It might become a challenging task in future to grow rice by transplanting due to the climatic change, water and labor scarcities. Direct-sown rice (DSR) is emerging as a resource-conserving and climate-smart alternative to transplanted rice (TPR). However, no specific variety has been bred for dry/direct-sown conditions. The present study was undertaken to decipher the molecular basis of genetic plasticity of rice under different planting methods. Comparative RNA-seq analysis revealed a number (6133) of genes exclusively up-regulated in Nagina-22 (N-22) leaf under DSR conditions, compared to that (3538) in IR64 leaf. Several genes up-regulated in N-22 were down-regulated in IR64. Genes for growth-regulation and nutrient-reservoir activities, transcription factors, translational machinery, carbohydrate metabolism, cell cycle/division, and chromatin organization/epigenetic modifications were considerably up-regulated in the leaf of N-22 under DSR conditions. Complementary effects of these factors in rendering genetic plasticity were confirmed by the agronomic/physiological performance of rice cultivar. Thus, growth-regulation/nutrient-reservoir activities, transcription factors, and translational machinery are important molecular factors responsible for the observed genetic plasticity/adaptability of Nagina-22 to different planting methods. This might help to develop molecular markers for DSR breeding, replacing TPR with DSR for better water-productivity, and minimizing greenhouse-gas emission necessary for negative emission agriculture.

## Introduction

Rice (*Oryza sativa* L.) cultivation, a staple food crop for a larger global population^1^, requires plenty of water for its continuous irrigation, particularly when grown by transplanting. Rice is primarily grown by transplantation, and > 75% of the rice is cultivated by transplanting world over^2^. However, several problems are associated with the cultivation of rice which includes scarcity of water, shortage of labor, and emission of greenhouse gases, etc. Transplanted rice (TPR) requires lots of water starting from preparation of land (puddling) till maturity (throughout the growing season) for frequent/continuous irrigation^3^. A larger proportion of water is lost from the rice field through evaporation and percolation, which result in very low productivity of water^4^. Moreover, the changing climatic conditions and increasing input costs threaten sustainable rice production in the future^5^. In addition, TPR is one of the major culprits of global greenhouse gas emissions responsible for > 11% emanation of anthropogenic gases (e.g. CH_4_)^6^. Alternate wetting–drying^7^, and dry/direct seeding of rice^8^ have been suggested to be the safer alternatives of TPR.

Direct sowing of rice might help to manage the water and labor scarcities as well as the increasing cost of cultivation^9^. Poor germination of seeds and stand establishment of direct-sown/dry-seeded rice (DSR) are some of the major constraints for achieving optimal crop growth and production; especially during water-deficiency stress^9^. Till date, no specific variety has been bred for dry-seeded/direct-sown conditions but the naturally adapted genotypes have been selected as DSR cultivars. *Indica* and *aus* rice has been reported to be more suitable for the direct-sown conditions with better yield potential than *japonica* rice^10,11^. However, high-yielding *indica* rice cultivars are not adapted to DSR conditions. Direct/dry-sowing of rice is generally practiced in rain-fed, drought-prone areas^12^, but higher infestation of weeds/nematodes are some of the major problems for the adoption of DSR^2,13^. A cultivar well-suited to DSR conditions must combine the adaptability of upland/aerobic rice and the high-yielding potential of lowland/anaerobic rice^14^.

DSR is adopted for only 28% of the areas under rice cultivation in India, while it is adopted for 45% of the areas in Korea, 47% of the areas in Vietnam, and for > 90% of the areas under rice cultivation in Malaysia, Sri Lanka, and the United States of America^15^. Mechanization of DSR not only saves water but also reduces labor requirement, emission of greenhouse gases, increases the farmer’s income with positive effects on soil health^15,16^. For DSR, seeds are directly sown in dry/non-puddled soil in the field^17^; thus, gaining popularity among the farmers because of the reduced requirement of water, labor, and lower cost of cultivation. The yield of DSR could become equivalent to that of TPR if irrigation and weeds/nematode infestation can be managed^18^. Therefore, having several disadvantages of TPR, the need of the day is to replace TPR with mechanized DSR. Replacing TPR with DSR would result in higher productivity of water towards the realization of an apt slogan “*Per Drop More Crop*” with the focus on saving/conserving water for ecological integrity^19^.

Nagina 22 (N-22), a tall, deep-rooted, drought and heat tolerant *aus* rice cultivar^20^, is suitable for DSR in the rain-fed areas where intermittent water-deficit stress is very common. Being a short-duration, drought, and heat-tolerant cultivar, it is well-suited for upland cultivation by direct sowing. In contrast, IR64 is a semi-dwarf, shallow-rooted, high-yielding rice cultivar developed primarily for irrigated/transplanted conditions^21–23^. It is sensitive to abiotic stresses and shows a considerable reduction in yield due to water-deficit stress^24^ at the reproductive stage. Under aerobic (upland/dry/direct-sown) conditions, its yield potential was reported to be significantly lower^25^.

DSR faces certain key challenges including reduced nutrient uptake, particularly nitrogen, phosphorus, iron, and zinc^26^. Under aerobic conditions, oxidation of ferrous (Fe^2+^, available form) to ferric (Fe^3+^, unavailable form) ion leads to iron deficiency in rice^27^. Lower nutrient uptake under direct-sown/dry-seeded conditions causes adverse effects on plant growth, tillering, leaf area index, photosynthesis, and grain filling^28^. Uniform seedling emergence is one of the crucial determinants of stand establishment and yield of DSR^13^. Vigorous seedlings and biomass accumulation at the vegetative stage are some of the important traits required for adaptation to DSR conditions^26^. Some of the major QTLs for traits like early seedling emergence, vegetative vigor, root architecture, plant height, and biomass production, which are important for DSR, have already been identified^26^. Such RING-H2 finger-containing ATL family proteins (ATL, Arabidopsis Tóxicos en Levadura) participate in defense responses, regulation of carbon/nitrogen metabolism during post-germinative seedling growth, regulation of cell-death during root development, endosperm development, and transition to flowering under short-day conditions^29^. While the stand establishment improves the adaptation of plants to DSR conditions^14^, better root system architecture (RSA) improves water and nutrients uptake from soil^30^. Although several nutrient-efficient, high-yielding progenies from multi-parent advanced generation inter-cross populations have been identified by Subedi et al.^14^, the candidate genes and their functions in adaptation to DSR conditions have not yet been explored.

Though several attempts have been made to analyze the transcriptome of rice to decipher the candidate genes for specific growth conditions, biotic, and abiotic stresses^31–34^, no report is available on transcriptome analysis to elucidate the genes/pathways involved in the adaptation of rice to DSR conditions. Therefore, an attempt was made here to decipher the genes/mechanisms/pathways responsible for genotypic plasticity of rice showing better performance under DSR conditions. Addressing the challenges of early/uniform emergence, better vegetative growth, efficient RSA, effective nutrient uptake under DSR conditions are necessary to develop rice varieties suitable for DSR. Dissecting the genetic/epigenetic basis of genotypic plasticity and developing high-yielding resource-efficient DSR variety using the modern biotechnological approaches have become the needs of the day. A better understanding of the molecular basis of RSA, nutrients use-efficiency, and stress tolerance might help developing rice varieties with better adaptability to DSR conditions. More importantly, this might pave the way for developing DSR cultivars to mitigate the effects of global climate change for better ecological efficiency.

## Results

### Morphophysiogical/agronomic performance of rice under different planting methods

The effects of planting methods on germination/seedling-vigor, growth and development of plant indicated a significant effect of dry/direct-sowing on germination-vigor (Supplementary Fig. S1). Nagina-22 showed significantly better germination-vigor, compared to that of IR64, when grown by dry/direct-sowing. Out of the 15 seeds sown by dry/direct-seeding, only nine germinating seeds/seedlings could be observed for IR64, while it was > 12 for N-22. Moreover, vigor of the N-22 seedling was observed to be better, with more number of surviving seedlings and better growth, compared to that of the IR64 seedlings grown by dry/direct-sowing (Supplementary Fig. S2). However, vigor of N-22 and IR64 seedlings was equally good when raised in the nursery (Supplementary Fig. S3). However, a difference in height of (N-22 taller) seedlings in the nursery was merely because of the genotypic variation.

A significant difference in the characteristic features of the root of the rice cultivars was observed when both were grown by direct-sowing. Root system of 30-day-old seedlings of IR64, when grown by direct-sowing, was less developed compared to that of the N-22. The total root length of N-22 was 32% more than that of IR64. The number of root tips in the case of N-22 (141) was significantly more than that of IR64 (124). Similarly, the root surface area and volume were significantly higher in the case of N-22 when it was grown by direct-sowing compared to that in the case of IR64 (Supplementary Fig. S4).

**Figure 1:**
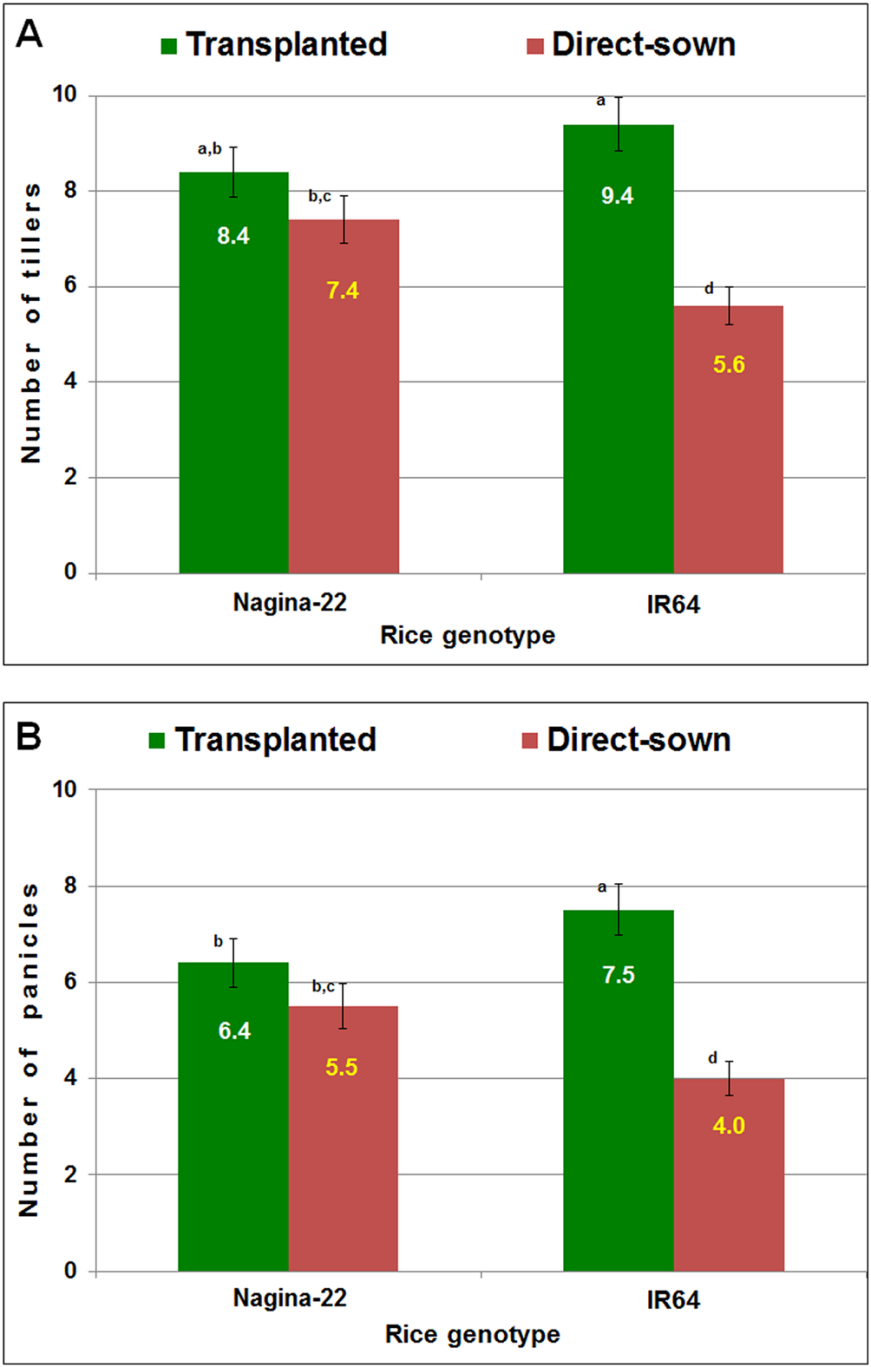
Performance of rice under transplanted and direct-sown conditions. (**A**) The number of tillers produced by each plant of Nagina-22 and IR64 under transplanted and direct-sown conditions, (**B**) the number of panicles produced by each plant of Nagina-22 and IR64 under transplanted and direct-sown conditions.

IR64, being a high-yielding rice cultivar, showed a higher number of tillers (9.4) per plant when grown by transplanting. A significant reduction in the number of tillers (5.6) per plant was observed when IR64 was grown by direct-sowing. When N-22 was grown by transplanting, the number of tillers (8.4) per plant was comparatively less than that of IR64. But the number of tillers (7.4) for direct-sown N-22 was comparatively higher than that observed (5.6) for IR64 grown by direct-sowing (Fig. 1A). Agronomic performance of the cultivars grown by different planting methods, as assessed based on the number of panicles per plant, indicated that IR64 possessed a higher number of panicles (7.5) per plant when grown by transplanting. However, a considerable reduction in the number of panicles (4.0) was observed when it was grown by direct-sowing. On the other hand, when N-22 was grown by transplanting, the number of panicles (6.4) per plant was comparatively lesser than that (7.5) of IR64. More importantly, the number of panicles of N-22 (5.5) grown by direct sowing was significantly more than that observed (4.0) for direct-sown IR64 (Fig. 1B, Supplementary Fig. S5). The difference between the numbers of panicles of N-22 grown by transplanting and direct sowing was not significant.

A significant increase in P content in roots of N-22 was observed when grown by direct-sowing. On the contrary, P content in roots of IR64 was lesser when grown under DSR conditions (Supplementary Fig. S6A). Similarly, the P content of leaf of N-22 grown by direct-sowing was higher compared to that grown by transplanting. A lower P content in the leaf of IR64 was observed when it was grown under DSR conditions (Supplementary Fig. S6B). Higher P content in the panicle of N-22, but lower in the panicles of IR64, grown by direct-sowing (compared to that grown by transplanting) indicated better nutrient uptake by N-22 under DSR conditions (Supplementary Fig. S6C).

### Transcriptome library preparation, sequencing and mapping on reference genome

To have a comprehensive understanding of the genes/pathways involved in acclimatization of rice to direct-sown conditions, leaf and root tissues from the rice cultivars grown by different planting methods were compared by RNA-seq analysis. A total of 16 libraries for two tissues from N-22 and IR64 cultivars grown until the panicle initiation stage by direct-sowing and transplanting were prepared successfully in two replications for whole transcriptome analysis. A total of 446 million reads with an average of 27 million reads for each sample were generated. Reference-based mapping of RNA-seq data on the rice reference genome (TIGR v7) using HiSat2 and Stringtie showed a very good (~ 87% uniquely mapped reads) mapping efficiency (Table 1).

**Table 1:** Summary of RNA-seq data mapping statistics.

Sample ID	Replication	Description	Total reads	Trimmed reads	Mapping efficiency (%)
ILT_1	1	IR64, leaf, transplanted	27386167	23114022	94
ILT_2	2		25671834	22054135	90
ILD_1	1	IR64, leaf, direct-sown	25656359	23292473	84
ILD_2	2		23977864	21677964	87
IRT_1	1	IR64, root, transplanted	28328497	24851122	82
IRT_2	2		26234865	22568344	84
IRD_1	1	IR64, root, direct-sown	29001797	25332254	86
IRD_2	2		27569645	24452243	89
NLT_1	1	Nagina-22, leaf, transplanted	34310721	29693196	91
NLT_2	2		32013216	28189603	87
NLD_1	1	Nagina-22, leaf, direct-sown	28560495	24463753	82
NLD_2	2		25764058	21465336	87
NRT_1	1	Nagina-22, root, transplanted	30317813	28727297	92
NRT_2	2		28138716	24761352	89
NRD_1	1	Nagina-22, root, direct-sown	27273614	23083513	90
NRD_2	2		26256312	24526123	78

### Differential expression of genes under direct-sown condition

**Figure 2:**
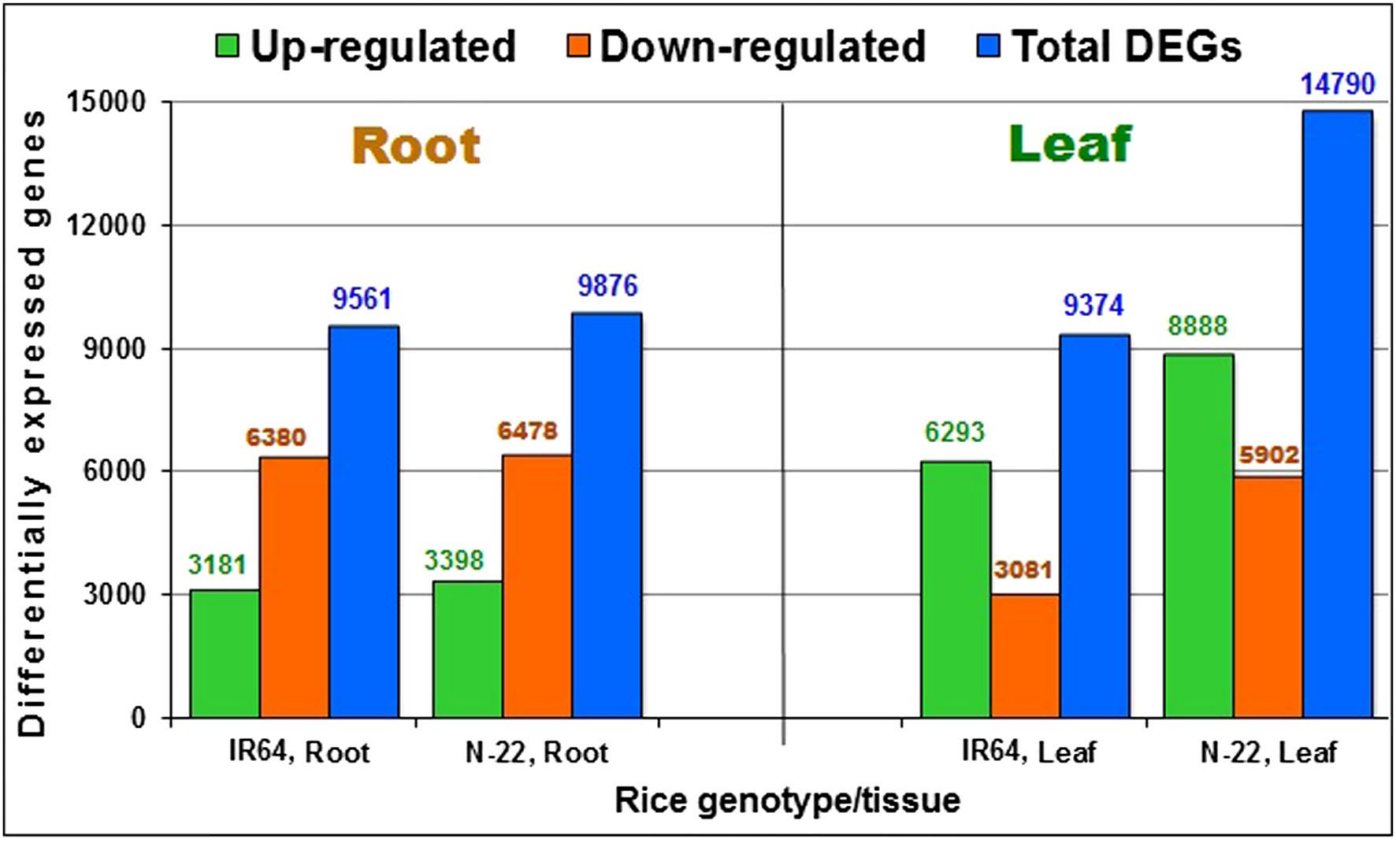
Differentially expressed genes (DEGs) in rice cultivars on direct-sowing over transplanting (control). Leaf and root tissues were collected at the panicle initiation stage of the plant for transcriptome analysis.

To decipher the genes/pathways responsible for the adaptation of rice to direct-sown conditions, comparative transcriptome analysis of leaf and root was performed over transplanting (control), which resulted in the identification of DEGs up- or down-regulated under direct-sown conditions. In roots of N-22 grown by direct-sowing, a total of 9876 DEGs with 3398 up-regulated and 6478 down-regulated genes were observed. In the roots of IR64 grown by direct-sowing, 9561 DEGs with 3181 up-regulated and 6380 down-regulated genes were identified. Moreover, leaves of N-22 grown by direct sowing showed 14790 DEGs comprising 8888 up-regulated and 5902 down-regulated genes. Similarly, leaves of IR64 grown by direct-sowing showed 9374 DEGs including 6293 up-regulated and 3081 down-regulated genes (Fig. 2).

**Figure 3:**
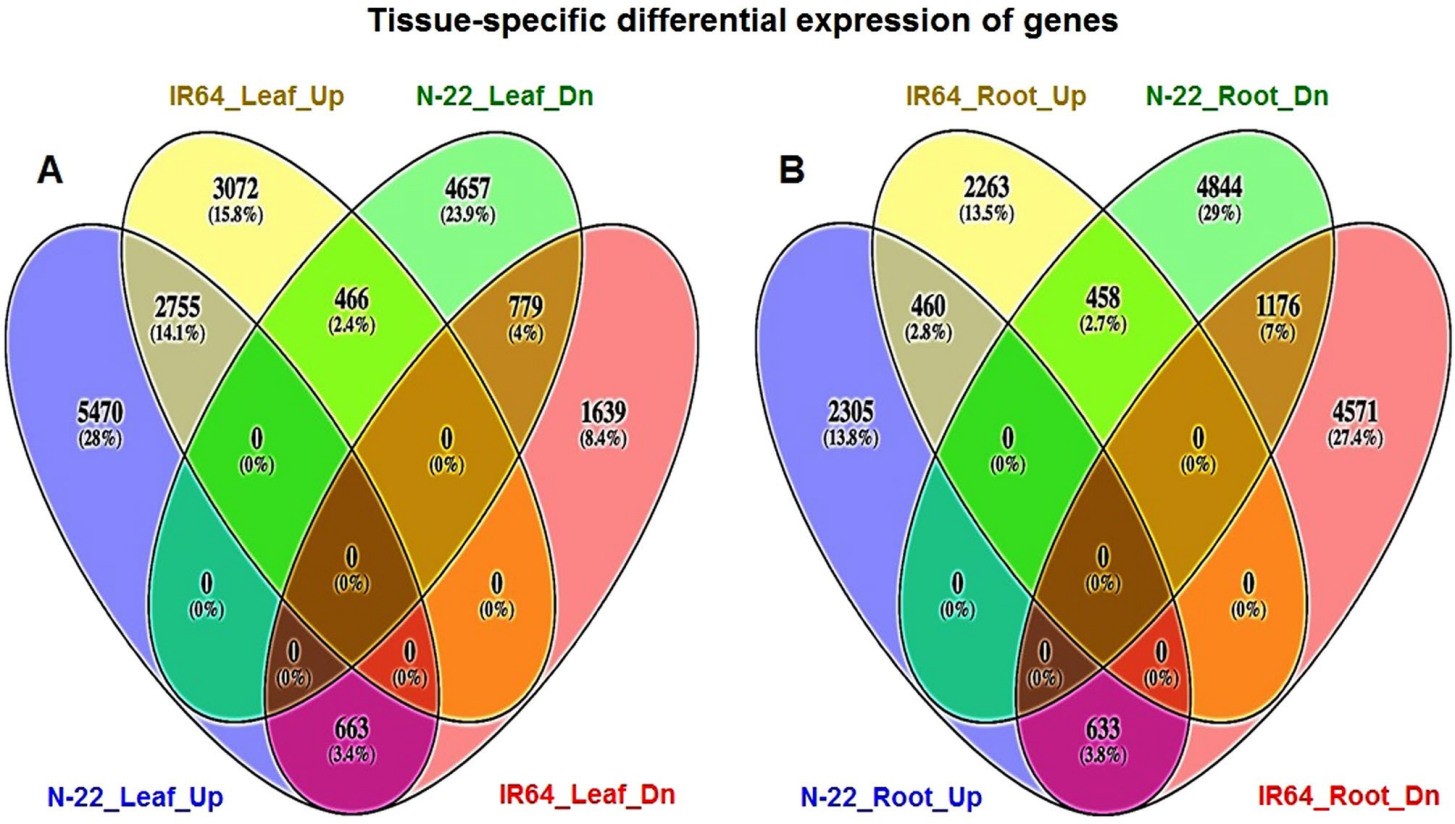
Tissue-specific differential expression of genes on direct-sowing. (**A**) Leaf, and (**B**) Root. Change in expression (Up = up-regulation, or Dn = down-regulation) was calculated based on the expression level of the genes on direct sowing (treatment) over transplanting (control).

A large number (6133) of genes were observed to be up-regulated exclusively in the leaf of N-22 on direct-sowing compared to that (3538) in the leaf of IR64 (Fig. 3A, Supplementary Table S2). Only 2755 genes were commonly up-regulated in the leaves of both the rice cultivars. The number of exclusively down-regulated genes (5123) in the leaf of N-22 was considerably higher than that (2302) in the leaf of IR64 (Fig. 3A). However, 663 genes up-regulated in the leaf of N-22 were observed to be down-regulated in the leaf of IR64. Moreover, 466 genes down-regulated in the leaf of N-22 were observed to be up-regulated in the leaf of IR64. Similarly, the number (2938) of genes observed to be up-regulated exclusively in the roots of N-22 on direct-sowing was comparatively higher than that (2721) in the roots of IR64 (Fig. 3B). Only 460 genes were commonly up-regulated in the roots of both the rice cultivars. The number of exclusively down-regulated genes (5302) in the roots of N-22 was higher than that (5204) in the roots of IR64. However, 633 genes up-regulated in the roots of N-22 were observed to be down-regulated in the root of IR64. Moreover, 458 genes down-regulated in the roots of N-22 were observed to be up-regulated in the roots of IR64 (Fig. 3B).

About 5737 genes were observed to be up-regulated exclusively in the leaf of IR64 on direct-sowing, while 2649 genes were down-regulated. Moreover, 2625 genes were up-regulated, while 5948 genes were down-regulated in the root of IR64. While 556 genes were commonly up-regulated in leaf and root of IR64 cultivar, only 432 genes were commonly down-regulated in leaf and root of IR64 (Supplementary Fig. S7A). Similarly, 8252 genes were up-regulated but 5458 genes were down-regulated in the leaf of N-22. About 2762 genes were up-regulated but 6034 genes were down-regulated in the roots of N-22. While 636 genes were commonly up-regulated in leaf and root of N-22 cultivar, 444 genes were commonly down-regulated in leaf and root of N-22 (Supplementary Fig. S7B).

### GO terms associated with acclimatization to direct-sown conditions

**Figure 4:**
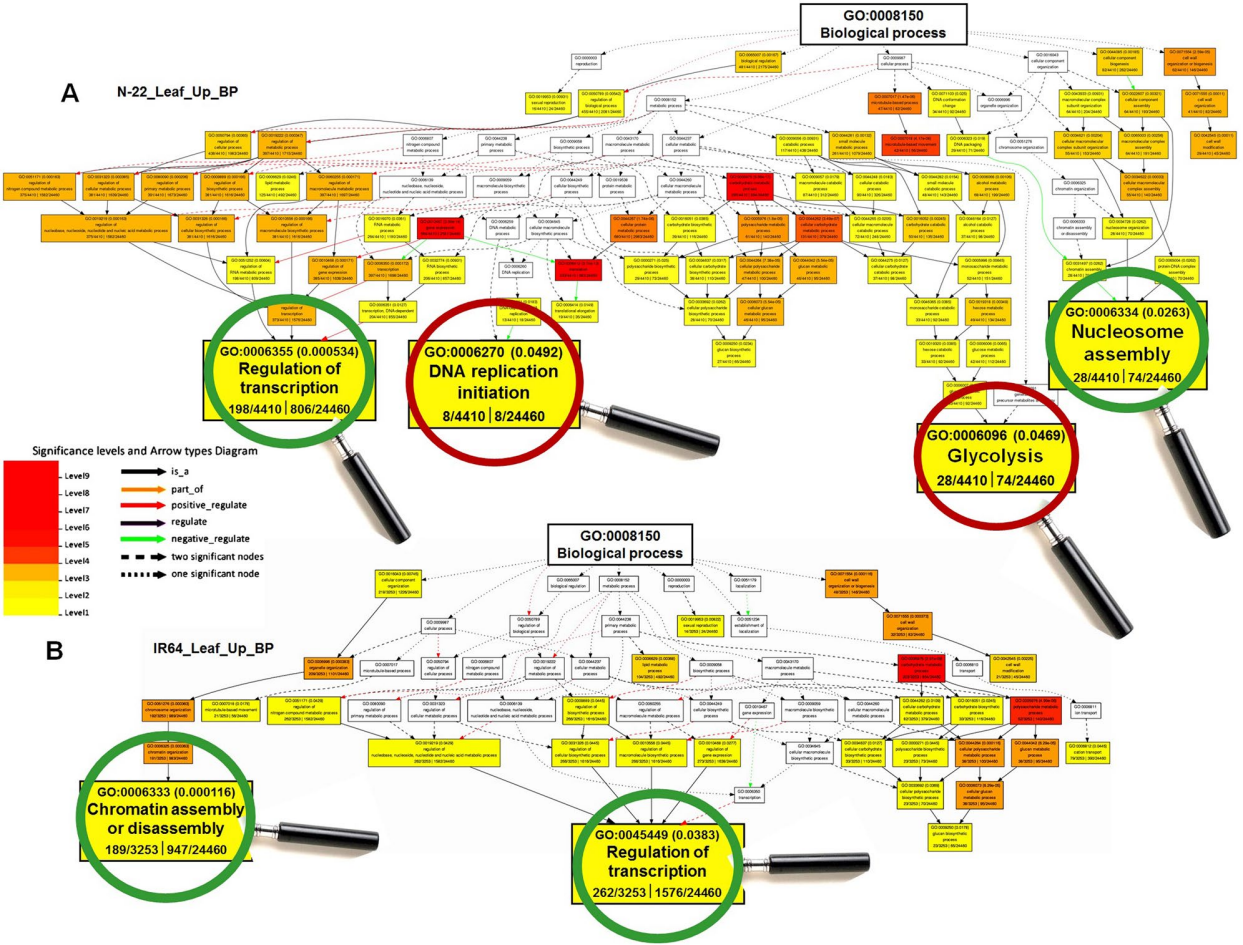
Gene ontology (GO) analysis of enriched biological processes under direct-sown [over transplanted (control)] conditions in the leaf of rice cultivars. (**A**) Over-represented GO terms in the leaves of Nagina 22, and (**B**) over-represented GO terms in the leaves of IR64 rice cultivar.

Analysis of GO terms associated with acclimatization to the DSR conditions revealed certain biological processes (BP), including gene expression, translation, carbohydrate metabolism, microtubule movement, nitrogen metabolism, and cell wall organization, to be significantly enriched (over-represented) in leaves of N-22, compared to that in the leaves of IR64. However, translation and glucose catabolism were exclusively enriched in the leaves of N-22 (Fig. 4). Similarly, several BP terms (post-translational protein modifications, regulation of transcription, isoprenoid biosynthesis, iron-sulfur cluster assembly, protein amino acid phosphorylation, photosynthetic light reaction, pollen-pistil interaction, programmed cell death, transmembrane transport, and metal ion transport) were under-represented in the leaves of N-22, compared to that in the leaves of IR64 (Supplementary Fig. S8).

**Figure 5:**
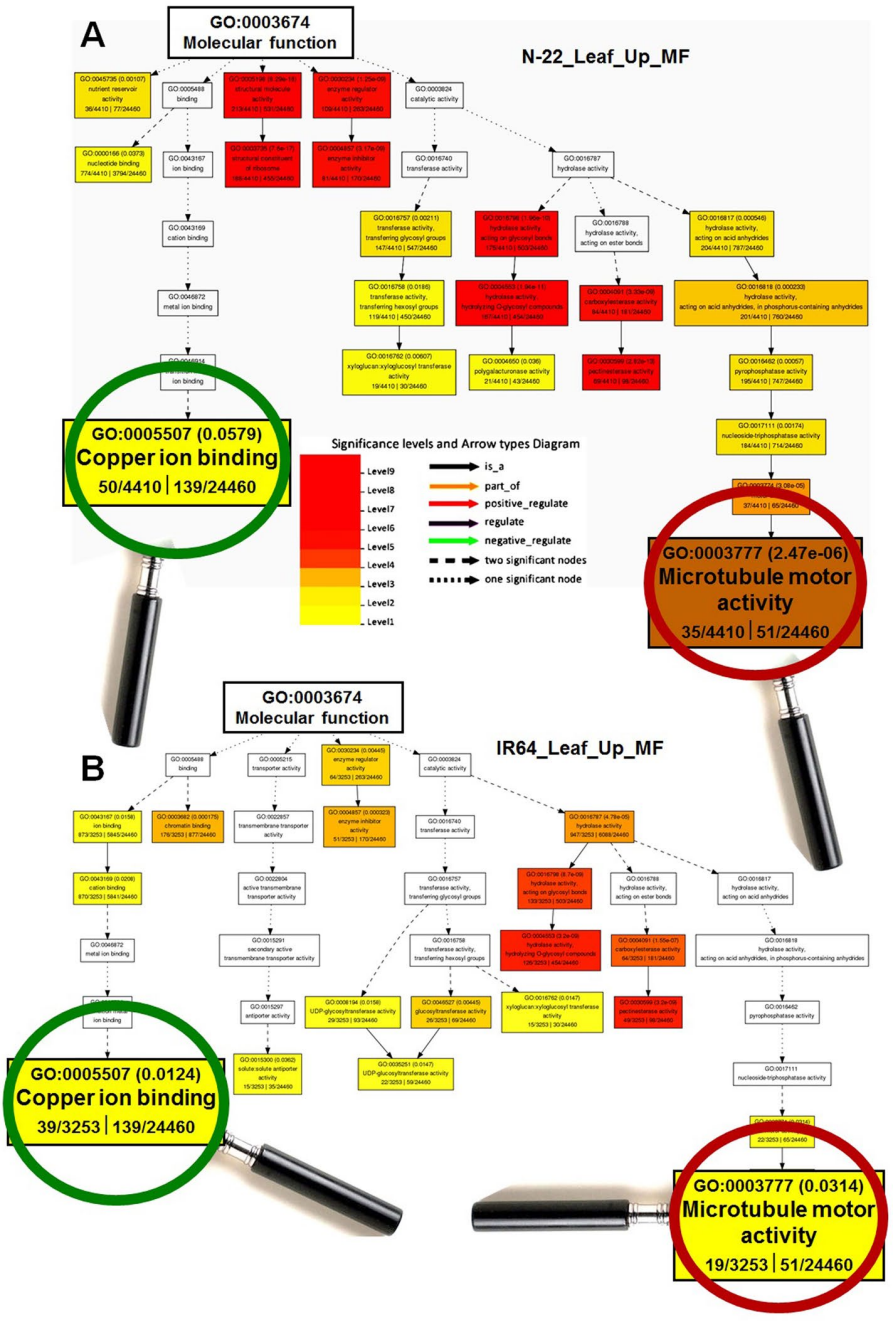
Gene ontology (GO) analysis of enriched molecular functions under direct-sown [over transplanted (control)] conditions in the leaf of rice cultivars. (**A**) Over-represented GO terms in the leaves of Nagina 22, and (**B**) over-represented GO terms in the leaves of IR 64 rice cultivar.

GO terms for molecular function (MF), including nutrient reservoir and structural constituents of ribosomes, enzyme inhibitor activity, pectinesterase activity, and microtubule motor activity, were significantly enriched in leaves of N-22, compared to that in the leaves of IR64 (Fig. 5). Similarly, MF terms for monooxygenase activity, protein serine/threonine- or tyrosine-kinase activity, heme-binding, ATP binding, and antiporter activity were under-represented in the leaves of N-22, compared to that in the leaves of IR64 (Supplementary Fig. S9).

GO terms for cellular components (CC) like cell wall, mitochondrial membrane, endoplasmic reticulum, ribosomal subunit, microtubule-associated complex, and nucleosome were over-represented in leaves of N-22, compared to that in the leaves of IR64 under DSR conditions (Supplementary Fig. S10). The CC terms for thylakoid membrane and oxygen-evolving complex were under-represented in the leaves of N-22, but no representation was observed in leaves of IR64 when grown under direct-sown conditions (Supplementary Fig. S11).

Moreover, analysis of the GO terms differentially presented in the contrasting rice cultivars on transplanting over direct-sowing (control) indicated certain biological processes (BP) like intracellular protein transport, signal transduction, cellular homeostasis, and histone modification to be significantly enriched (over-represented) in leaves, particularly in the case of IR64 (Supplementary Fig. S12). However, the GO terms like gene expression, translation, cellular amino acid metabolic process and nucleosome assembly were under-presented in leaves of IR64 on transplanting (Supplementary Fig. S13). However, many of the GO terms were similarly over-presented (Supplementary Fig. S14) or under-represented (Supplementary Fig. S15) in roots of both the cultivars on transplanting.

### Expression of genes for growth regulation related activities

Differential expression of the genes for growth regulation factors was observed in the leaf as well as in the root under DSR conditions. Twelve genes for growth regulation-related activities were observed to be > 2-fold (2.36–13.92-fold) up-regulated in the leaves of N-22 on direct sowing, compared to that observed on transplanting (Supplementary Table S3). Seven genes were observed to be more up-regulated in the leaves of IR64 on direct sowing compared to that observed in the leaves of N-22. LOC_Os04g48510 and LOC_Os04g24190 showed root-specific expression in the rice cultivars, with 6.23-fold up-regulated expression of LOC_Os04g24190 in roots of N-22 (Supplementary Table S3).

Interestingly, the growth regulating factors showing up-regulated expression in leaf of N-22 under direct-sown conditions were performing their functions in roots on transplanting, particularly in IR64 (Supplementary Table S4). Such differential expression of the genes for growth regulating factors in the rice plants grown by different planting methods was entirely different, particularly with respect to the tissue involved.

### Expression of genes for nutrient reservoir activities under direct-sown conditions

Several genes for glutelin (LOC_Os02g14600, LOC_Os02g15070, LOC_Os02g16830), prolamin (LOC_Os06g31070, LOC_Os06g31060, LOC_Os07g10580, LOC_Os07g10570), cupin (LOC_Os01g50900, LOC_Os08g09000), phosphate transporters (LOC_Os03g05610, LOC_Os03g05640, LOC_Os07g31430), iron transport (LOC_Os04g45520, LOC_Os11g05010, LOC_Os08g05720), magnesium transporter (LOC_Os04g35160), sulfate transporter (LOC_Os01g52130), and nitrate transporter (LOC_Os02g38230) were considerably up-regulated in leaves of N-22 under DSR conditions. Some of these genes were also up-regulated in the roots of N-22 compared to that in the roots of IR64. Inorganic phosphate transporter (*OsPHT1;9*, LOC_Os06g21920) was up-regulated exclusively in roots with more up-regulation in N-22 (Supplementary Table S5).

**Figure 6:**
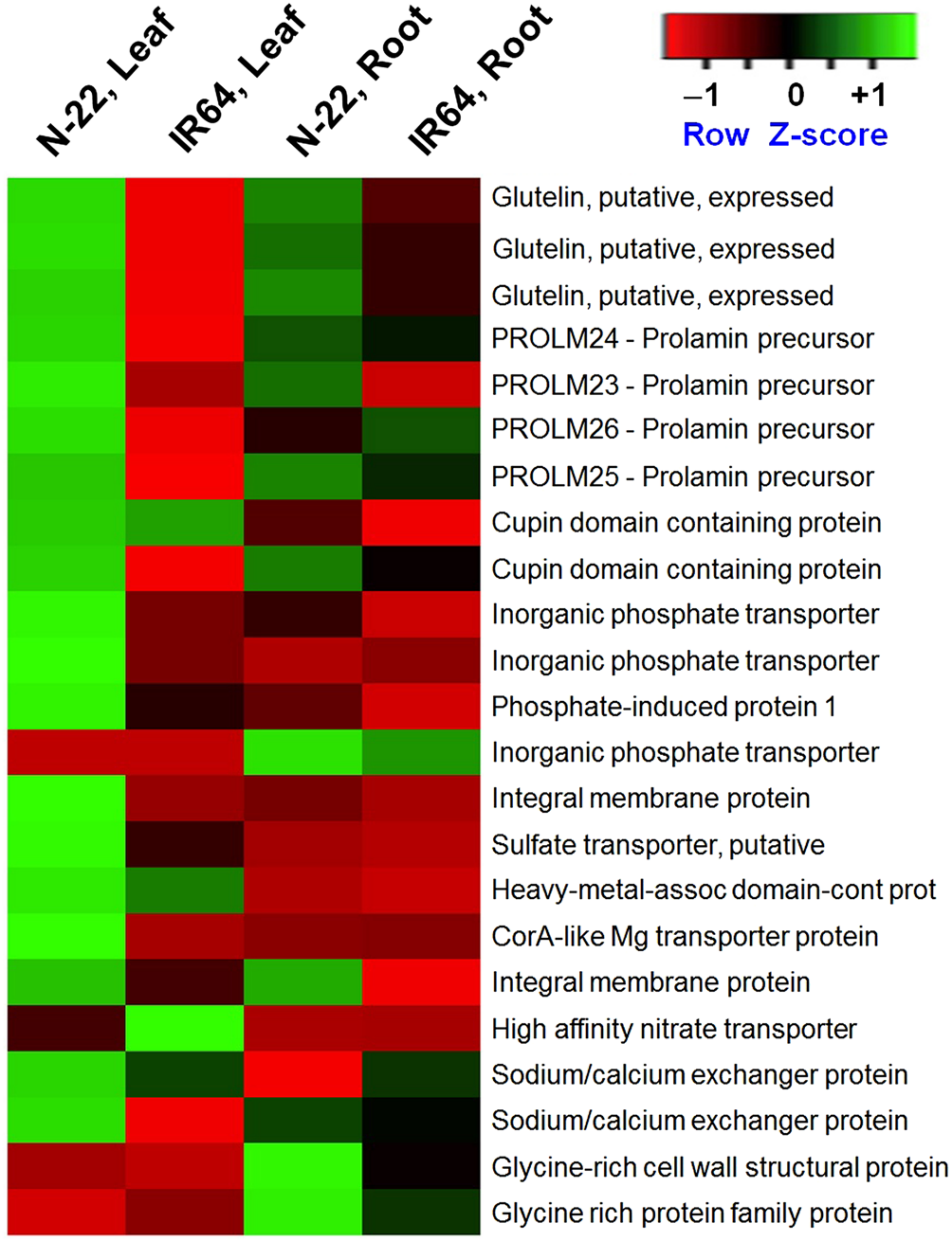
Heat map showing differential expression of some of the differentially expressed genes associated with nutrient reservoir activities under direct-sown conditions in the leaves and roots of rice cultivars.

However, a high-affinity nitrate transporter (LOC_Os02g38230) was observed to be more than 2 times (5.29-fold) up-regulated in the leaves of IR64 compared to that observed (2.2-fold) in the leaves of N-22. Most of the genes for glutelin and prolamin, the two major nutrient storage proteins, were considerably up-regulated in the leaves of N-22 but down-regulated in the leaves of IR64. Some of the genes for prolamin (LOC_Os06g31060), phosphate transporter (LOC_Os03g05610), iron uptake (LOC_Os03g01290, LOC_Os08g05720), sulphate transporter (LOC_Os01g52130), and magnesium transporter (LOC_Os04g35160) were observed to be highly up-regulated in the leaves of N-22 but not expressed in the leaves of IR64 (Fig. 6, Supplementary Table S5). Moreover, some of the genes like glycine-rich proteins (LOC_Os04g56030, LOC_Os04g56040) were up-regulated in the roots of N-22 under DSR conditions.

Interestingly, the expression level of several genes associated with the nutrient reservoir activities was observed to be up-regulated in roots of the rice cultivars under transplanted conditions (over direct-sown condition) (Supplementary Fig. 16B). Variation in the gene expression and their role in genetic plasticity of can be clearly estimated from their expression in shoot (under direct-sown conditions) and root (under transplanted conditions) (Supplementary Fig. 16).

### Expression of transcription factors on direct sowing of rice

**Figure 7:**
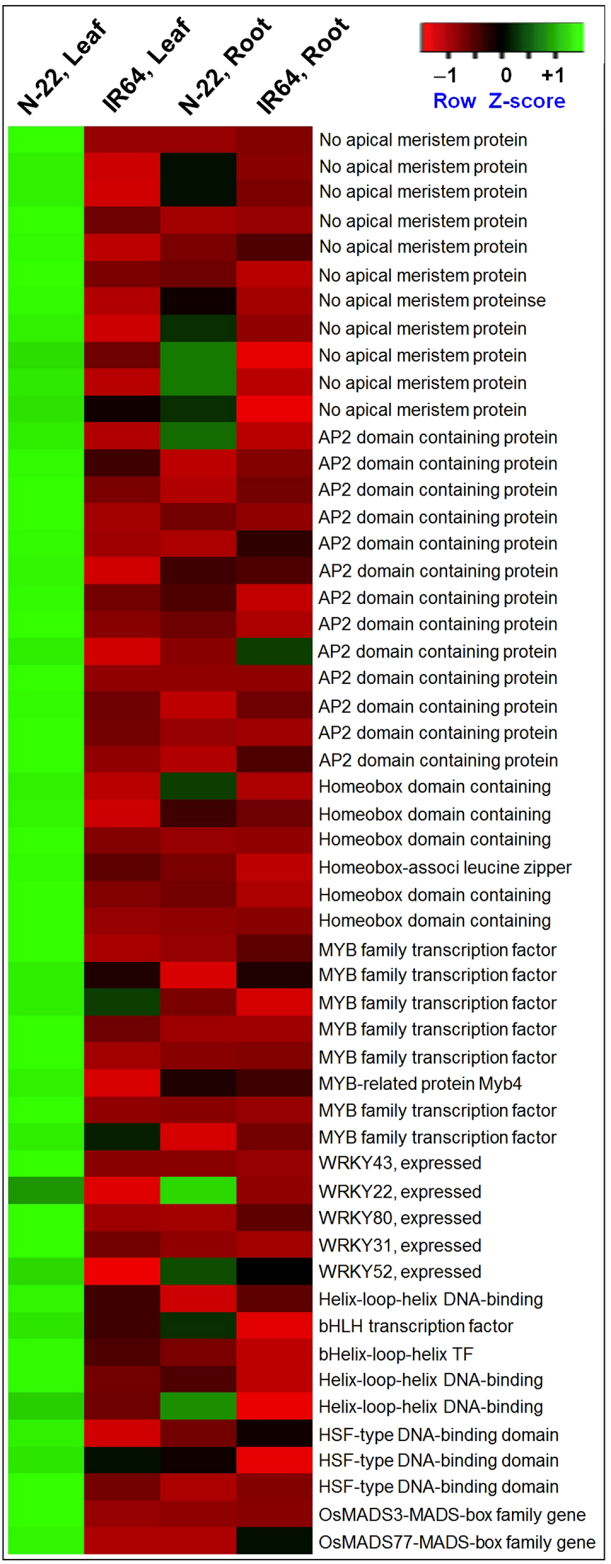
Heat map showing differential expression of some of the differentially expressed genes for transcription factors under direct-sown conditions in the leaves and roots of rice cultivars.

Transcription factors (TFs) of 8 different families were considerably up-regulated in leaves of N-22 on direct-sowing. More than 18 ‘No apical meristem’ family TFs were > 2-fold up-regulated in the leaves of N-22 but their expression in the leaves of IR64 was not affected under direct-sown conditions. Similarly, TFs of AP2, homeobox domain-containing, MYB, WRKY, bHLH, HSF, and MADS-box families were significantly up-regulated in the leaves of N-22 under DSR conditions but little or not affected in the leaves of IR64. Even in the roots of N-22, some of these TFs were up-regulated but they were either not affected or down-regulated in the roots of IR64. TFs like No apical meristem (LOC_Os05g25960), AP2 (LOC_Os03g07940), and MYB (LOC_Os01g63680) were exclusively expressed in N-22 but their expression was not detected in IR64 under direct-sown conditions. Some of the No apical meristem (LOC_Os11g3136, LOC_Os07g09740, LOC_Os03g01870, LOC_Os06g36480, LOC_Os07g27340), AP2 (LOC_Os06g42990, LOC_Os04g56150, LOC_Os03g08460), Homeobox domain-containing (LOC_Os03g10210), MYB (LOC_Os01g74020, LOC_Os09g36730, LOC_Os04g38740, LOC_Os09g26170), WRKY (LOC_Os05g49210, LOC_Os06g30860, LOC_Os02g47060), bHLH (LOC_Os03g03000, LOC_Os07g09590, LOC_Os03g55220, LOC_Os03g42100), HSF (LOC_Os09g28200, LOC_Os07g44690), and MADS-box (LOC_Os01g10504) TFs were up-regulated exclusively in the leaves of N-22 under direct-sown conditions (Fig. 7, Supplementary Table S6).

However, under transplanted conditions many of the TFs showed up-regulation (over their expression under direct-sown conditions), particularly in roots of the rice cultivars (Supplementary Table S7, Supplementary Fig. 17B). But the TFs up-regulated in N-22 were different from those up-regulated in IR64. Interestingly, a large number of these TFs were considerably up-regulated in N-22 under direct-sown conditions (Supplementary Fig. 17A).

The comparative transcriptome analysis revealed 21 NF-Y TFs (involved in the regulation of embryogenesis, flowering time, chloroplast biogenesis, seed germination, and stress tolerance) comprising 6 NF-YA, 6 NF-YB, and 9 NF-YC subunits differentially expressed under DSR conditions. Remarkably, 20 of these genes were up-regulated (up to 16.28-fold) in the leaves of N-22 compared to that in the leaves of IR64 under direct-sown conditions. While one of the NF-YC (LOC_Os01g24460) factors expressed only in N-22 (leaf and root) cultivar, LOC_Os02g49410 (NF-YB) was observed to be expressed exclusively in the leaves of N-22 under direct-sown conditions (Supplementary Table S8).

### Differential expression of translational machinery under direct-sown conditions

A total of 71 DEGs encoding for various components of translation machinery were identified under DSR conditions. Most of the genes showed up-regulated expression in leaves of N-22, compared to that in the leaves of IR64, including those coding for various translation initiation and elongation factors, and the ribosomal subunit (Supplementary Table S7). Some of the genes (LOC_Os05g22716, LOC_Os04g16834, LOC_Os07g17770, LOC_Os07g28710) showed comparatively more up-regulated expression in the roots of N-22 under DSR conditions. While LOC_Os07g28710 and LOC_Os08g06560 were up-regulated in N-22 (leaf and root), LOC_Os07g14750 was expressed exclusively in the leaves of N-22 under DSR conditions (Supplementary Table S9).

### Differential expression of genes for proteolytic activities on direct-sowing

A higher level of transcripts for 14 genes involved in proteolytic activities was observed in leaves of IR64 compared to that in the leaves of N-22 under DSR conditions. While 5 genes were not expressed in the leaves of N-22, 2 genes showed considerable down-regulation under DSR conditions. The expression level of all the 14 genes was significantly higher in the leaves of IR64, while it was least affected in the root of IR64 as well as in the leaf and root of N-22. The subtilisin, carboxypeptidase, SKP1-like, and ubiquitin fusion degradation proteins, as well as aspartic proteinase, were 2.23–10.5-folds up-regulated in the leaves of IR64 (Supplementary Table S10).

### Differential expression of genes for carbohydrate metabolic process

The expression level of genes involved in the carbohydrate metabolic process was observed to be more up-regulated in leaves of N-22 compared to that in the leaves of IR64. A large number of genes for carbohydrate biosynthetic process, particularly polysaccharide (glucan) biosynthetic process, were highly expressed in the leaves of N-22 compared to that in the leaves of IR64 under DSR conditions. Moreover, the number and level of expression of genes involved in the photosynthetic process, particularly photosynthetic electron transport in photosystem II, were observed to be higher and down-regulated in the leaves of N-22 under direct-sown conditions (Supplementary Fig. S11). The expression level of genes involved in glycolysis and Krebs cycle were up-regulated in leaves of N-22 compared to that observed in IR64 under direct-sown conditions. Interestingly, the gene (LOC_Os09g30240) for rate-limiting enzyme in the glycolysis pathway (Phosphofructokinase) was observed to be highly (8.55-fold) up-regulated in the leaves of N-22 under direct-sown conditions (Supplementary Table S11).

### Differential expression of genes for chromatin assembly/epigenetic modifications

Under direct-sown conditions, more than 33 genes associated with chromatin/nucleosome assembly in leaves of N-22 compared to that in leaf and root of IR64. Some of the genes responsible for DNA modification [encoding for methyl-binding protein (LOC_Os05g33554), C-5 cytosine-specific DNA methylase (LOC_Os07g08500)], histone modification [JmjC domain-containing protein, acetyltransferase, SNF2/7 family N-terminal domain-containing protein, Methyltransferase domain-containing protein, and H3 lysine-9 specific SUVH1] were highly up-regulated in the leaves of N-22, while marginally up-regulated, not affected, or down-regulated in the roots of N-22, leaves as well as roots of IR64 under direct-sown conditions. About 16 genes coding for core histone H2A/H2B/H3/H4 proteins or histone H3 proteins were 2–10-fold up-regulated in the leaves of N-22, with LOC_Os12g22650 expressed exclusively in the leaves of N-22, while LOC_Os12g22680 and LOC_Os06g06510 up-regulated exclusively in N-22 (leaf and root) under direct-sown conditions (Supplementary Table S13).

Interestingly, the gene for chromatin assembly showing up-regulated expression in leaf of N-22 under direct-sown conditions could perform their functions in roots on transplanting, particularly in N-22 (Supplementary Table S14). However, variation in the expression of the genes for epigenetic modifications was not so evident under transplanted conditions that could be observed under direct-sown conditions.

### Differential expression of some other important genes under direct-sown conditions

In addition to a large number of DEGs of various activities/pathways (Supplementary Fig. S18), some other groups of genes playing crucial roles in plant growth at the early stage, stand establishment, tolerance to abiotic and biotic stress, and related activities were observed to be significantly up-regulated in the leaf of N-22 under DSR conditions (Supplementary Table S15). Nine genes of TOR (target of rapamycin) signaling pathway coding for serine/threonine-protein kinases (e.g. LOC_Os05g36050, LOC_Os05g35760, LOC_Os11g06140, LOC_Os05g35770, LOC_Os02g34430, LOC_Os03g17550, LOC_Os07g44360, LOC_Os05g33080, and LOC_Os10g01560) involved in the phosphorylation of eukaryotic initiation factor 3 were observed to be considerably (> 6–11-fold) up-regulated in leaves of N-22. At least five genes for late embryogenesis abundant (LEA) protein (e.g. LOC_Os05g46480, LOC_Os07g17120, LOC_Os06g21910, LOC_Os04g49980, LOC_Os08g23870) responsible for early plant growth and development were observed to be considerably (> 6–10-fold) up-regulated in leaves of N-22. Similarly, the genes for certain transporters like ABC transporters (e.g. LOC_Os08g05710, LOC_Os08g05690), peptide transporter (LOC_Os03g01290), and DNA replication/repair (e.g. LOC_Os11g04954, LOC_Os02g35450, LOC_Os02g56130, LOC_Os08g05840, LOC_Os10g29660) were significantly up-regulated in the leaf of N-22 under DSR conditions. More importantly, the genes responsible for better root system architecture (LOC_Os02g05060), drought tolerance (e.g. LOC_Os01g73770, LOC_Os04g48330, LOC_Os06g03670, LOC_Os02g45450, LOC_Os04g48350), and tolerance to root-knot nematode (e.g. LOC_Os03g03510, LOC_Os12g02200) were significantly up-regulated in roots of N-22 compared to that in roots of IR64 under DSR conditions. Some of these genes (e.g. LOC_Os04g57610, LOC_Os02g05060, LOC_Os02g05050, LOC_Os01g73770, LOC_Os06g03670, LOC_Os06g03750, LOC_Os05g14240, LOC_Os03g03510, LOC_Os01g52050) were also significantly up-regulated in the leaf of N-22 compared to that in the leaf of IR64 (Supplementary Table S15).

### Validation of RNA-seq data by RT-qPCR analysis

**Figure 8:**
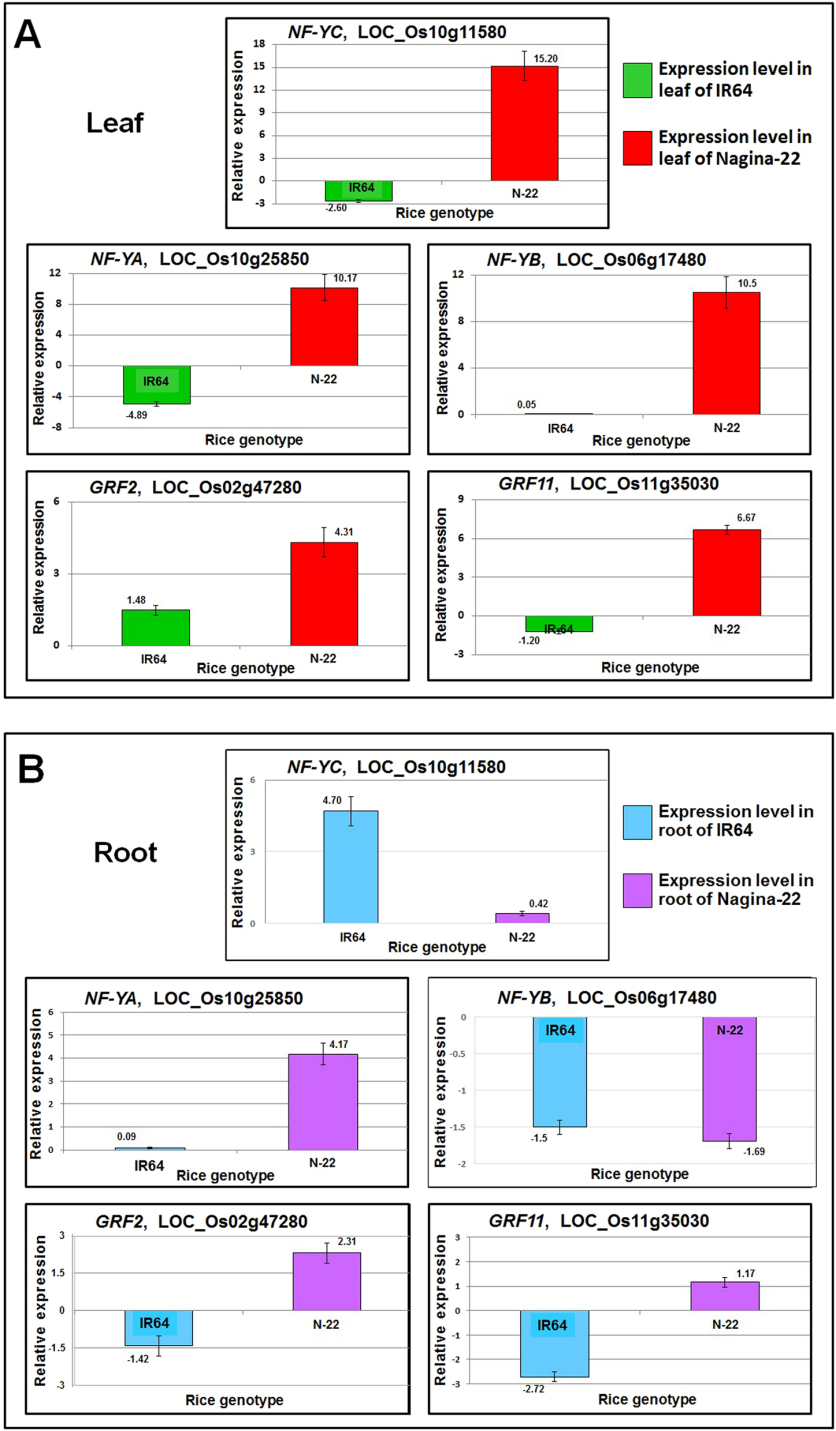
RT-qPCR validation of five randomly selected differentially expressed genes. cDNA was prepared from the total RNA isolated from leaf and root tissues of Nagina 22 (N-22) and IR64 collected at the reproductive stage of plants grown by transplanting (control) or direct-sowing (treatment). Data represent the mean ± SD (n = 6).

The expression profile of genes observed in transcriptome data was validated by RT-qPCR expression analysis of five randomly selected DEGs in leaf and root of plants in response to different methods of planting. The results demonstrated a similar pattern of expression of the genes as detected by the transcriptome analysis (Fig. 8). Thus, RT-qPCR analysis confirmed the trustworthiness of the transcriptome data.

## Discussion

Cultivation of rice by dry/direct-sowing is an environment-friendly and resource-saving practice, as it supports rice production with limited availability of water/labor and mitigates the emission of greenhouse gases. Efforts are being made to map the changes in metabolic activities and identify the key traits favorable for DSR. These have resulted in recognition of traits like the root system architecture, nutrient reservoir activities, uniform emergence, early vigor, abiotic/biotic stress tolerance, lodging resistance, etc. to be advantageous for DSR. To delineate the genes/pathways responsible for acclimatization of rice to direct-sown conditions, a comparative transcriptome analysis of rice cultivars was performed.

Dry/direct-sowing showed a significantly better germination-vigor of N-22 compared to that of IR64 (Supplementary Fig. S1). Early germination and emergence of seedlings (at 4 days after sowing) was recorded in the case of N-22, while no germination could be noted for IR64 (Supplementary Fig. S1). This indicated the early emergence ability of N-22 even at lower soil moisture content. N-22, being an abiotic stress tolerant genotype, showed significantly better root characteristic features when grown by dry/direct-sowing. The longer and more proliferated roots of N-22 seedling, compared to that of the IR64, must be one of the factors responsible for the better performance of N-22 under dry/direct-sown conditions (Supplementary Fig. S4).

The comparative RNA-seq analysis indicated only a minor (~ 3%) difference in the number of DEGs between the roots of the rice cultivars, while the number of DEGs was considerably (> 41%) higher between the leaves of the rice cultivars (Fig. 2). Moreover, the up-regulated expression of genes in the leaf of N-22 might be responsible for the genotypic plasticity, which played important role in the acclimatization of this cultivar to DSR conditions (Fig. 3, Supplementary Fig. S1). Top 100 genes identified to be considerably (> 10-fold) up-regulated in the leaf of N-22 under DSR conditions and responsible for better growth and development included the growth-regulating factors, nutrient reservoir activities, various transcription factors, translational machinery, and chromatin organization (Supplementary Table S2). Interestingly, the GO enrichment analysis revealed some of the GO terms to be enriched with such considerably up-regulated genes in the leaf of N-22. These genes/pathways might be responsible for making N-22 a suitable cultivar for the direct-sown conditions.

Growth regulating factors (GRFs), observed to be more up-regulated in leaves of N-22 compared to that in the leaves of IR64 under direct-sown condition (Supplementary Table S3), are plant-specific factors that play role in controlling growth and abiotic stress tolerance^35,36^. These might help to manage better growth and development of N-22 under DSR conditions. Interestingly, LOC_Os09g32320, LOC_Os02g06400, and LOC_Os11g35030 were ~ 2.5 times more up-regulated in leaves of N-22 compared to that in IR64. Moreover, a root-specific GRF (LOC_Os04g24190) [6.23-fold up-regulated in N-22, but down-regulated (− 1.32-fold) in IR64], might be responsible for better RSA and performance of N-22 under DSR conditions (Supplementary Table S3). Only a few genes (LOC_Os03g51970, LOC_Os02g53690, LOC_Os02g45570, and LOC_Os12g29980) were marginally more up-regulated in the leaves of IR64, compared to that in the leaves of N-22, under DSR conditions. GRFs were reported to play important role in the early growth and development of plants and regulate leaf development, stem elongation, and root growth^37–39^. GRFs were reported to be strongly expressed in buds, leaves, flowers, and promote the growth and development of rice by regulating cell proliferation in the actively growing tissues^40^. *OsGRF6* (LOC_Os03g51970) was reported to regulate the growth and development of inflorescence in rice^41^. RNA interference transgenic lines for *OsGRF* (LOC_Os02g53690) resulted in reduced growth, development, smaller leaves, and delayed heading^42^. Thus, the possibility of increasing the expression level of GRFs in N-22 under DSR conditions favors its genotypic plasticity and candidature to be a suitable cultivar for DSR. Up-regulated expression of the genes for growth regulating factors in roots of the rice plants grown by different planting methods, particularly in IR64 (Supplementary Table S4), indicates the reason why these cultivars perform better under transplanted conditions. This also indicate the role of leaves in protecting the rice plants under direct-sown conditions, while roots help the plants to perform better under transplanted conditions.

Many genes for nutrient storage proteins, such as glutelin and prolamin, are considerably up-regulated in the leaf of N-22 but down-regulated/less expressed in IR64, and a similar pattern in roots also (Supplementary Table S5), suggest that N-22 has better genotypic plasticity to be a suitable cultivar for DSR. Glutelin is one of the major storage proteins which accounts for 50% of the total seed proteins^43^. Uptake of zinc (Zn) and iron (Fe) is also influenced by environmental factors such as soil texture, soil moisture, availability of nutrients in the soil (content and its form), and abiotic stresses^44^. In DSR, dry/aerobic conditions often accelerate the oxidation of organic matters, convert available ferrous (Fe^2+^) to unavailable ferric (Fe^3+^) form, and restrict Zn availability to plants^27,45^. Thus, Zn and Fe deficiency in DSR becomes a common problem^46^. Several genes related to nutrient uptake/transport such as phosphate transporters (LOC_Os03g05610, LOC_Os03g05640, LOC_Os07g31430) and iron transporters (LOC_Os04g45520, LOC_Os11g05010, LOC_Os08g05720) were considerably up-regulated in leaves of N-22 under DSR conditions, thereby support its better performance (Fig. 6). Moreover, an inorganic phosphate transporter (*OsPHT1;9*, LOC_Os06g21920) was up-regulated exclusively in roots of N-22 under DSR conditions. Expression of this particular transporter was recently reported to be root-specific in a tolerant genotype under P deficiency stress^47^. Phosphorus not only works as an activator for > 60 enzymes but also regulates the water content and reduces the adverse effects of salts in the plant. Nutrient reservoir activities play important roles in genetic plasticity of N-22 to perform better under both direct-sown (by up-regulated expression of the genes in leaf) and transplanted (by up-regulated expression of the genes in root) conditions (Supplementary Fig. 16).

A family of TFs such as WRKY, bZIP, MYB, AP2/EREBP, and NAC play important roles in controlling growth/developmental processes, cellular morphogenesis, and responses to environmental stress^48^. We observed considerably up-regulated expression of TFs of eight important families in leaves of N-22 under DSR conditions. Eighteen NAC family TF namely no apical meristem (NAM), playing important role in the shoot apical meristem development^49^, were > 2-fold up-regulated in leaves of N-22 under DSR conditions which must be responsible for early establishment of N-22 under DSR conditions. Similarly, different TF families like AP2, homeobox domain, MYB, WRKY, bHLH, HSF, and MADS-box were considerably up-regulated in leaves of N-22 under DSR conditions (Fig. 7, Supplementary Table S6). While AP2/ERF regulates agronomic traits like plant growth and defense responses through phytohormone biosynthesis and signaling^49,50^], WRKY plays roles in plant development, biotic and abiotic stress tolerance^51–53^. Heat shock proteins (molecular chaperones) and heat shock factors (HSFs) regulate cellular homeostasis and promote survival under stressful conditions^54,55^. Over-expression of *OsHsfA7* was reported earlier to improve salt and drought tolerance in rice^56^. Up-regulated expression of some of the genes for TF in roots of the rice cultivars grown by transplanting (Supplementary Table S7, Supplementary Fig. 17) indicates the reason for their better performance under transplanted conditions. However, the complementary effects up-regulated expression of many of these TFs makes N-22 better performer even under adverse/varying environmental conditions generally observed in the DSR filed. The data (Supplementary Fig. 17) clearly indicate the difference in genetic plasticity of these two cultivars for their ability to grow and perform under direct-sown and transplanted conditions.

NF-Y family members were reported to have a strong correlation with water-deficiency stress tolerance in many plant species. Our observation on 20 NF-Y family TFs (comprising of 5 NF-YA, 6 NF-YB, and 9 NF-YC) considerably up-regulated in leaves of N-22 might be responsible for its better adaptability under DSR conditions. Exclusively expressed LOC_Os01g24460 (> 13-fold up-regulated), LOC_Os02g49410 (> 12-fold up-regulated) in leaves of N-22 under DSR conditions, along with LOC_Os10g11580 (> 16-fold up-regulated), and considerably up-regulated expression of LOC_Os10g25850 (> 12-fold) in N-22 (Supplementary Table S8) must be the major contributors in making N-22 suitable for DSR. NF-Y has been reported to be involved in the regulation of embryogenesis, flowering time, chloroplast biogenesis, seed germination, and stress tolerance^57^. The importance of NF-Y in stress tolerance was demonstrated by overexpressing a citrus *NF-YA5* in transgenic tobacco showing improved dehydration tolerance^58^.

Translation is one of the important steps in eukaryotic gene expression/protein synthesis, which is classically divided into three steps: initiation, elongation, and termination. Our observations on the up-regulated expression of several genes encoding for the components of translational machinery in N-22 under DSR conditions indicate better regulation of translation process in this cultivar. Regulation of translation is a central aspect of plant growth, development, and tolerance to environmental stress^59^. Since translation is a most energy‐demanding process, fine-tuning the regulation of this process is necessary not only for the faster production of the desired proteins but also to adjust the level of protein synthesis according to the actual demand. Stage and cell‐specific global changes in the translation process were reported during plant growth and development^60,61^. Translation initiation is the first key step in controlling protein synthesis, and eukaryotic initiation factors (eIFs) play important role in the formation of initiation complex, recruitment of 40S ribosomal subunit, scanning for the start codon (AUG), formation of the ternary complex, and other processes^62,63^.

Ribosomal function is dynamic in nature, particularly under fluctuating environmental conditions. Ribosomal proteins (RPs) are not only essential constituents of the ribosome but also required for decoding mRNA. Growing evidence indicates that RPs play important roles in translational regulation particularly under abiotic stresses and developmental processes^64–66^. In response to the iron and phosphorus deficiencies, global induction of RPs was reported in Arabidopsis^67^, which indicates that alteration in the composition of ribosomes affects translational efficiency. The composition of RPs was reported to change in response to the developmental and environmental cues in plants^68^, enabling the ribosome to regulate gene expression. Moreover, post-translational modifications (acetylation, methylation, and methylthiolation) of RPs might also be environment-dependent^69,70^. Significantly up-regulated expression of several ribosomal proteins (e.g. LOC_Os11g04070, LOC_Os07g14750, LOC_Os08g06560) in leaves of N-22 under DSR conditions (Supplementary Table S9) supports the involvement of RPs in translational regulation in N-22 under the fluctuating environmental conditions generally observed for DSR.

In plants, the cap-binding complex [composed of cap-binding protein (eIF4E), scaffolding protein (eIF4G) and RNA-binding proteins (RBP, Pumilio)] checks for mRNA integrity, stimulates the initiation of translation and promotes recycling of 40S ribosomal subunit^71^. The eIF4A (helicase), in association with a cofactor eIF4B, remove the secondary structure of mRNA for the formation of 43S pre-initiation complex (PIC) comprising of 40S ribosome subunit, eIF1, eIF3, eIF5, and interact with a ternary complex (comprised of GTP-binding protein eIF2, and the Met-tRNA^fmet^ initiator). Our observation on significantly up-regulated expression of eIF2 (LOC_Os02g39350, LOC_Os03g21550, LOC_Os12g07740), eIF4 (LOC_Os07g36940, LOC_Os04g40400, LOC_Os02g38220), and RBP (e.g. LOC_Os12g30520, LOC_Os12g38590, LOC_Os11g37090) in leaves of N-22 under DSR conditions indicates increased formation of PIC. The significantly up-regulated expression of methionyl-tRNA synthetase (LOC_Os01g60660), responsible for charging tRNA^fmet^, indicates the preparedness of translational machinery. Up-regulated expression (~ 1.5-fold) of eIF4G (LOC_Os07g36940) was observed in leaves of N-22 under DSR conditions. Similarly, eIF4B (LOC_Os04g40400, LOC_Os02g38220), and eIF4A/DEAD-box ATP-dependent RNA helicase (LOC_Os06g48750/LOC_Os02g05330) were significantly up-regulated in leaves of N-22 (Supplementary Table S9). These observations corroborate with findings of the earlier researchers.

The eIF3, involved in many steps of initiation and brings PIC to the cap structure, is required for scanning of the start codon (AUG), inhibiting the premature association of 60S ribosomal subunit, and reinitation of the translation process^72^. The mRNA associated with all the initiation factors makes 43S PIC and joining of 60S ribosomal subunit complete the formation of initiation complex^73^. Our observation on the significantly up-regulated expression of eIF1 (LOC_Os01g09890), eIF3 (e.g. LOC_Os02g02990, LOC_Os07g12110, LOC_Os05g49150, LOC_Os05g01450, LOC_Os04g30780, LOC_Os10g41960, LOC_Os07g03230), eIF5 (LOC_Os05g51500, LOC_Os12g32240, LOC_Os07g02210), and 60S ribosomal proteins in the leaves of N-22 under DSR conditions indicates the increased formation of the initiation complex. The eIF6 (LOC_Os07g44620) playing important role in regulation of 80S ribosomal assembly (Supplementary Fig. S13A) was considerably up-regulated in N-22 under DSR conditions (Supplementary Table S7). Moreover, polyA-binding proteins were also significantly up-regulated in leaves of N-22 under DSR conditions, which again support the hypothesis of improved translational efficiency in N-22. Elongation factors play an important role in translation by mediating the translocation step in peptide chain elongation. In *Medicago falcate*, eEF2 was reported to play role in cold tolerance through regulating the synthesis of proteins^74^. Our observations on the significantly up-regulated expression of several elongation factors (e.g. LOC_Os06g37440, LOC_Os07g42300, LOC_Os07g46750) in leaves of N-22 under DSR conditions (Supplementary Table S9) corroborate with the above findings.

Recent findings indicate the role of stress, hormones, and metabolic signals in regulating the target of rapamycin (TOR, an evolutionarily conserved serine/threonine kinase) signal-transduction pathway which integrates nutritional status, cell cycle, growth, and development with the environmental stress. Thus, the TOR pathway integrates nutrient-, energy-, and stress-related signals to adjust protein biosynthesis^75^. Functions of the TOR pathway in regulating translational process in mammals are known, but it is yet elusive in plants. Phosphorylation of RPs through the TOR signaling pathway was reported to be one of the mechanisms of complex translational regulation system^63,71^. Hence, the TOR signaling pathway (a highly conserved master coordinator) connects RPs with stress, energy, and nutritional status in regulating protein biosynthesis^76^. Higher expression of *TOR* in embryo and endosperm of Arabidopsis, and *tor* null mutant showing growth-arrest at embryonic stages indicate that TOR signaling is involved in early growth and development of plant^76^. We observed considerably up-regulated expression of TOR pathway genes like serine/threonine-protein kinase (e.g. LOC_Os05g36050, LOC_Os05g35760, LOC_Os11g06140, LOC_Os05g35770, LOC_Os02g34430, LOC_Os03g17550, LOC_Os07g44360; Supplementary Table S15) involved in phosphorylation of eIF3. TOR signaling pathway was also reported to be involved in the selective translation of a subset of transcripts^71^. A protein kinase CK2 (Casein kinase 2), a ubiquitous Ser/Thr kinase evolutionarily conserved in eukaryotes, has been extensively studied in mammalian systems which phosphorylates > 300 substrates, including transmembrane proteins, in developmental, environmental, and hormonal response pathways^77^. Under stressful conditions, CK2 phosphorylates eIF3 (eIF3h) with the help of TOR (Supplementary Fig. S17B). Thus, translational regulation during environmental stress is mediated through TOR signaling pathway^63^. Manipulation of TOR kinase activity resulted in the changes in growth/development of Arabidopsis from embryogenesis to senescence^78–81^. Therefore, plant TOR kinase acts as a caretaker in estimating the nutritional status of the plant and accordingly regulates the development of the root system^76^.

Protein degradation is one of the important regulatory processes that enable cells to respond quickly to intracellular signals and environmental conditions by augmenting the synthesis of key proteins. Ubiquitin/26S proteasome system causes ubiquitination of protein leading to its degradation in eukaryotes^82^. A considerably higher transcript level of genes involved in proteolytic activities observed in leaves of IR64 under DSR conditions, compared to that in N-22, might be responsible for excessive proteolysis of some of the key proteins/enzymes required for optimal growth and development under the prevailing environmental conditions on direct sowing. Subtilisin, carboxypeptidase, SKP1-like, ubiquitin fusion degradation protein, and aspartic proteinase were more than 3–10-folds up-regulated in leaves of IR64 (Supplementary Table S10).

In plants, glycolysis and gluconeogenesis play a central role in cellular metabolism and provide unique abilities to respond dynamically to the changing environmental conditions throughout their life^83^. Both these pathways involve nearly reverse biochemical reactions, and most of the enzymes catalyze the reversible reactions^84,85^. Glucose-6-phosphate isomerase catalyzes the interconversion of glucose-6-phosphate and fructose-6-phosphate which is a reversible reaction^86^. Triosephosphate isomerase is involved in sugar metabolism and glycolytic synthesis of ATP^87^. Pyruvate kinase, 6-phosphofructokinase, hexokinase, and other enzymes of glycolysis were observed to be more up-regulated in leaves of N-22 compared to that in IR64 (Supplementary Table S9). Under an unfavorable environment, plant cells bypass ATP-dependent biochemical reactions of sugar metabolism using alternate Pi-independent enzymes. The genes coding for the enzymes involved in such glycolytic bypass showed more up-regulation in leaves of N-22. The first bypass event catalyzed by PPi-dependent phosphofructokinase (PPi-PFK, LOC_Os09g30240), converting the fructose-6 phosphate to fructose 1, 6-bisphosphate without consumption of ATP, was ~ 8.55-fold up-regulated in the leaves of N-22 (Supplementary Table S11). All of these indicate that better glycolysis and gluconeogenesis processes improve the ability of N-22 to perform under DSR conditions.

Differential expression of genes encoding the cell cycle-related proteins/enzymes, and considerably higher expression under DSR conditions in leaves of N-22 (Supplementary Table S12) must be responsible for better performance of N-22 on DSR cultivation. The cyclin-dependent kinase (CDK) inhibitors/assembly-factor/regulatory-subunit might be helpful in better coordination between cell division and plant growth needed for the proper development under DSR conditions^88^. CDKs play important role in modulating transcription and cell division in response to the extra- and intra-cellular cues^89^. Coordinated interaction of the mitotic cell cycle and cell expansion is necessary for plant growth under specific conditions^90^. Significantly higher up-regulated expression of other genes associated with mitotic cell division and related activities such as tubulin-like filamentous temperature-sensitive protein Z (tubulin/FtsZ) domain-containing proteins, mitotic spindle checkpoint protein, Nuf2 family protein in leaves of N-22 must be responsible for better adaptability of this cultivar under DSR conditions. Tubulin/FtsZ family is a group of conserved GTP-binding proteins closely related to cell division, organ formation, and plant development as a major component of the cytoskeleton, particularly for cytokinesis in chloroplast division in photosynthetic eukaryotes^91^. Moreover, the genes involved in DNA replication/repair (e.g. LOC_Os02g56130, LOC_Os08g05840, LOC_Os08g06620, LOC_Os11g04954, LOC_Os02g35450) were significantly up-regulated in leaves of N-22 (Supplementary Table S15). These might be necessary for the desired pace of cell division required for the appropriate growth and development of plants under such environmental conditions.

DNA methylation and histone modification are important epigenetic factors responsible for reprogramming gene expression under changing environmental conditions. Histone proteins being important components of chromatin structure, numerous histone-modifying enzymes help to modulate gene expression under environmental stresses^92,93^. Different chromatin remodelers including CHD, ISWI, and SWI/SNF act upon chromatin under environmental stresses to modulate its transcriptionally inactive/active state^94^. Gene expression is also regulated by biochemical modifications of chromatin and DNA methylation. Chromatin remodeling and DNA methylation may result in a highly condensed and tightly coiled chromatin complex often associated with alterations in gene expression to facilitate plant growth and development under a challenging environment^95^. Histone acetyltransferase and deacetylase have been reported to play important role in stress responses in plants^96–99^. Our observations on the significantly up-regulated expression of the genes associated with DNA methylation (LOC_Os05g33554, LOC_Os07g08500), histone modification (LOC_Os09g31380, LOC_Os03g05710, LOC_Os02g38300, LOC_Os05g41172), histone H3 protein (LOC_Os12g22680, LOC_Os06g06510), core histone proteins (LOC_Os06g06480, LOC_Os05g02300), NAP domain-containing proteins (LOC_Os04g38620), etc. in leaves of N-22 (Supplementary Table S13) indicate the involvement of epigenetic machinery in acclimatization of this cultivar to DSR conditions.

Growing evidence indicates that γ-tubulin not only functions in microtubule nucleation in transcription but also in DNA damage response and chromatin remodeling^100^. Along with the bundling and aggregating capacity, γ-tubulin filaments perform complex scaffolding and sequestration functions^101^. Our findings of significantly up-regulated expression of Tubulin/FtsZ domain-containing proteins in leaves of N-22 under DSR conditions (Supplementary Table S13) suggest important roles of tubulin proteins in chromatin remodeling. The gene for chromatin assembly/epigenetic modifications which showed up-regulated expression in leaf of N-22 under direct-sown conditions did not show important role in modulating gene expression under transplanted conditions (Supplementary Table S14).

**Figure 9:**
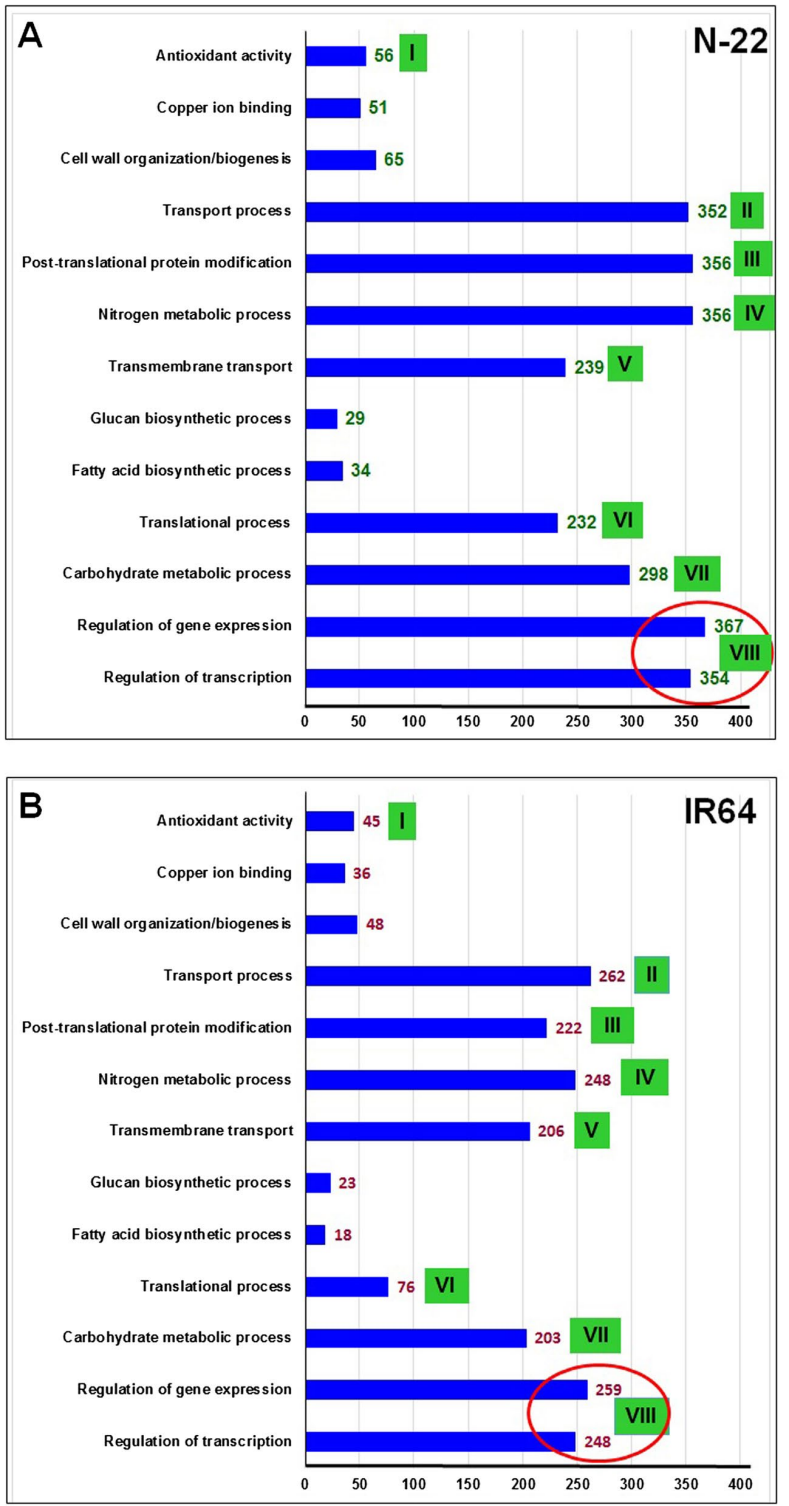
Differentially enriched GO terms and the number of respective genes up-regulated in the leaves of (**A**) Nagina-22 (N-22) and (**B**) IR64 rice cultivars grown by the different methods of planting (transplanting, control; direct-sowing, treatment). For comparative evaluation of N-22 and IR64, some of the important/similar GO terms have been numbered (I–VIII) and/or encircled.

GO enrichment analysis revealed that regulation of gene expression, transcription, and translational processes were significantly enriched with a significantly higher number of up-regulated genes in leaves of N-22 (Fig. 9A), which is necessary for the stand establishment. In addition, nitrogen and carbohydrate metabolic processes, transport process, post-translational protein modification, and antioxidant activity were also enriched in leaves of N-22 with a higher number of up-regulated genes for these GO terms. These must be responsible for the early vigor of the plants needed for crop establishment under such environmental conditions. More importantly, these GO terms were under-symbolized with a comparatively lesser number of up-regulated genes in leaves of IR64 (Fig. 9B).

Moreover, the gene for nutrient transport (e.g. LOC_Os08g05710, OC_Os08g05690) and early growth and development of plants (e.g. LOC_Os07g17120, LOC_Os06g21910, LOC_Os04g49980) were significantly up-regulated in leaves of N-22, which might be responsible for a better seedling vigor and early establishment of the crop. Significantly up-regulated expression of genes for RSA (e.g. LOC_Os04g57610, LOC_Os02g05060), water-deficit stress tolerance (e.g. LOC_Os01g73770, LOC_Os04g48330, LOC_Os06g03670), and root-knot nematode tolerance genes (e.g. LOC_Os03g03510, LOC_Os12g02200) in roots of N-22 under DSR conditions (Supplementary Table S12) must be responsible for better crop establishment under the environment. Interestingly, some of the retrotransposon/transposon genes (e.g. LOC_Os07g34550, LOC_Os12g27770, LOC_Os09g32930), particularly those coding for the proteins associated with metal ion/nutrient reservoir activities and cell cycle/division, considerably up-regulated in leaves of N-22 under DSR conditions must have important roles in genotypic plasticity of this cultivar.

Complementing effects of the above-mentioned molecular factors for genotypic plasticity could be confirmed at three different developmental stages of the rice plant. The survival rate and vigor of N-22 seedlings under DSR conditions were significantly more than that of IR64 seedlings (Supplementary Fig. S2). These findings corroborate with the expression level of five *LEA* genes (LOC_Os05g46480, LOC_Os07g17120, LOC_Os06g21910, LOC_Os04g49980, LOC_Os08g23870) which were 7–10-fold up-regulated in the leaf of N-22 under DSR conditions (Supplementary Table S15). At the vegetative stage, the effects of molecular factors could be confirmed by the number of tillers, which could be maintained (compared to that under TPR conditions) by N-22 under DSR conditions but a significant reduction in the number was observed in the case of IR64 when grown under DSR conditions (Fig. 1A). This indicates better vegetative vigor of N-22 even under DSR conditions. More importantly, a significant reduction in the number of panicles was observed in the case of IR64 when grown under DSR conditions (Fig. 1B), causing ~ 40% reduction in the yielding potential (Supplementary Fig. S5). However, the reduction in the number of panicles of N-22 was not significant (Fig. 1B, Supplementary Fig. S5) on dry/direct-sowing, which resulted in just a 15% reduction in the yielding potential. This confirms complementary effects of up-regulated expression of the genes for tolerance to abiotic (drought) and biotic (root-knot nematode) stresses (Supplementary Table S15) that the plants might have experienced under DSR conditions.

The importance of nutrient uptake/transport/assimilation machinery under DSR conditions in the rice cultivars was confirmed by the estimation of P content in different tissues of the plants. Due to up-regulated expression of phosphate transporters in roots of N-22 (Supplementary Table S5), particularly the root-specific transporter PHT1;9, the P content in roots of N-22 under DSR conditions was significantly higher than that observed under TPR conditions (Supplementary Fig. S6A). Because of the considerably up-regulated expression of phosphate transporters in the leaf of N-22, the Pi gets mobilized to different parts of the plant, which was evident from the P content in leaf and panicle (Supplementary Fig. S6B,C). The decreased availability/mobility of P in the aerial parts of IR64 affects its growth and development under DSR conditions. Significantly reduced P content in leaf and panicle of IR64 indicates lower uptake and lesser mobilization of P in IR64 and validates the down-regulated expression of phosphate transporters in IR64 under DSR conditions. These findings corroborate with the expression level of molecular factors observed in N-22 providing it better genotypic plasticity to cope with the varying environmental conditions faced by rice plants grown by different planting methods. Our observations on physiological and agronomic performances of the rice cultivar under TPR and DSR conditions are in agreement with the findings of Zhao et al.^25^, and Novoa and Loomis^28^. These findings support the complementary effects of growth-regulating factors, nutrient reservoirs, transcription factors, translational machinery, and carbohydrate metabolism, etc., which helped N-22 to perform better even under DSR conditions.

## Conclusions

DSR is an environment-friendly and resource-saving strategy that supports the concept of ‘producing more from less’ by saving water and mitigating the emission of greenhouse gases. There are certain ecological advantages of adopting DSR over TPR; however, more intensive efforts are needed to address the concerns in its adoption. The varieties developed for TPR conditions show considerable (~ 40%) yield reduction under DSR conditions; hence, identification of donors/QTLs/genes for various DSR-suited traits like early uniform emergence, vegetative vigor, better nutrient uptake/use-efficiency, higher grain yield, etc. become necessary for the DSR variety improvement program. Although QTLs for DSR-suited traits have been reported^14,26^, their interactions with different genetic backgrounds and environments have not yet been studied^12^. Interestingly, our observation on the genes associated with various DSR-suited traits located on different QTLs (Supplementary Table S16) and their expression level in shoot and roots of the rice cultivars indicated that many of such genes were comparatively more up-regulated in N-22, particularly in roots (Supplementary Table S17). Among these, RING-H2 finger-containing ATL family proteins and Homeobox domain-containing proteins were observed to be more important. The ATL family proteins have been reported to participate in various DSR-suited traits like defense responses, regulation of carbon/nitrogen metabolism during post-germinative seedling growth, regulation of cell-death during root development, transition to flowering under short-day conditions, and endosperm development^29^. Our findings indicate important roles of certain growth-regulating factors, nutrient reservoir activities, transcription factors, translational machinery, and carbohydrate metabolism, besides many other genes/pathways, in making N-22 cultivar better adapted to dry/direct-sown conditions. Understanding the ways a single protein kinase (e.g. TOR) acts as a master regulator to coordinate numerous cellular activities might help improving plant productivity even under unfavorable environmental conditions. Further research would be required to validate the candidature of the above-mentioned molecular factors (using the strategy like knockout of the gene) in providing genotypic plasticity and better adaptability to DSR conditions. Moreover, understanding the epigenetic and epitranscriptomic regulation of gene expression under environmental stresses^102^ would also be required. These might help improving rice varieties for dry/direct-sowing, mitigating the effects of global climate change, particularly the erratic rain-fall, emission of greenhouse gases for ecological integrity.

## Materials and methods

### Plant materials and growth conditions

Mature seeds of two rice cultivars (Nagina-22 and IR64) were used for raising the plants. Initially, both the rice cultivars were grown by direct-sowing and transplanting continuously for four generations (2016–2019). Then, the rice cultivars were grown once again by direct-sowing and transplanting (2020) to be utilized in the present study. For TPR, seedlings were raised in nurseries, followed by uprooting and transplanting of 28-days-old seedlings in 12″ pots filled with puddled soil. For DSR, mature seeds were directly sown in un-irrigated soil (having soil moisture content ~ 50% less than the control) at 2 cm depth in 12″ pots. The plants were grown in a net-house under natural conditions during the *Kharif* season (July–October) at the experimental farm of ICAR-Indian Agricultural Research Institute, New Delhi, India. Pots for the TPR were frequently irrigated (on an alternate day) with tap water, while the DSR pots were provided with life-saving irrigation only in absence of the seasonal rainfall. Leaf and root tissues were collected at the flowering/panicle initiation stage using liquid nitrogen in nine biological replicates for molecular analyses. The experimental research and field studies on plants (the cultivated rice genotypes) comply with the relevant institutional, national, and international guidelines and legislation.

### RNA isolation, cDNA library preparation and sequencing

Total RNA was isolated using the TRIzol method from six biological replicates of leaf and root tissues. About 500 mg tissue was ground into a fine powder using liquid nitrogen, and the powdered tissue was transferred to DEPC-treated 1.5 ml Eppendorf tube containing 1.0 ml of TRIzol reagent. The contents were mixed vigorously for 1 min and then incubated at 25 °C for 5 min. Chloroform (200 µl) was then added, mixed, and incubated at 25 °C for 5 min. The content was then centrifuged at 12,000 rpm for 15 min. The upper aqueous phase was carefully removed (avoiding the intake of interface) and transferred to an RNase-free 1.5 ml Eppendorf tube, an equal volume of isopropanol was added and mixed well by slowly inverting the tubes. The tubes were incubated overnight at − 80 °C, and then centrifuged at 14,000 rpm for 30 min. The pellet was washed with 1.0 ml of 75% ethanol by centrifugation at 12,000 rpm for 15 min. The pellet was then air-dried for 5 min, and the RNA pellet was dissolved in 30 μl of DEPC-treated water. The total RNAs was treated with DNase using DNase I kit (Qiagen, Germany) following the manufacturer’s instructions. To quantify the RNAs, OD value was recorded at 260 and 280 nm (for A_260_/A_280_ ratio) using NanoDrop (Thermo Scientific). To check the purity of the RNAs, denaturing agarose (1.2%) gel electrophoresis was performed, and the integrity of the RNAs was checked using Qubit 4 (IQ Assay Kit).

The isolated RNAs were pooled in two groups (3 + 3) to prepare two replications of the isolates RNAs for each of the leaf and root samples. A total of 16 cDNA libraries [2 cultivars, 2 planting methods (DSR and TPR), 2 tissues (leaf and root), and 2 replications] were prepared from the pooled RNA samples following the steps for mRNA enrichment, RNA fragmentation, first- and second-strand cDNA synthesis, purification, sequencing-adaptor ligation, and PCR amplification as suggested for the TruSeq RNA Sample Preparation kit (Illumina). The libraries were sequenced by PE-150 at the Illumina HiSeq 2500 platform. Raw sequence data were submitted to the NCBI SRA database under the SRA ID: PRJNA805549, and used for detailed bioinformatic analyses.

### Quality check and RNA-seq data analysis

Raw data were checked for quality using FastQC 0.11.7 (http://www.bioinformatics.babraham.ac.uk/projects/fastqc). Adapter contamination and low-quality reads (< phred33) were removed using Trimmomatic 0.36^103^. The resultant high-quality reads were mapped to the rice reference genome available in the database (http://rice.plantbiology.msu.edu) using HISAT2 (2.1.0) pipeline^104^ and assembled using StringTie software to assemble unique transcript sequences. Read counts were generated from the alignment files using Feature Counts software (Subread package 1.6.2) with default parameters^105^ based on rice RAP-DB annotation gtf file version 1.0.38. The number of mapped clean reads for each gene was counted and normalized into the reads per kilobase per million (RPKM) value. The differentially expressed genes (DEGs) were analyzed using the DESeq2 (V 1.20.0) package of the R program^106^. Threshold levels of FDR ≤ 0.1, *p* < 0.05, and log_2_ FC ≥  ± 2 were used to judge the significance of differences.

### Gene ontology analysis

Gene ontology (GO) enrichment analysis for the DEGs was performed using AgriGO v2 software, which helped in the identification of enriched/over-represented GO terms, as described by Kumar et al.^47^ earlier. The background list of genes and GO annotations were extracted from the RGAP database.

### Validation of DEGs by RT-qPCR

To validate the results obtained from transcriptome analysis, the expression level of some of the randomly selected genes (from the groups that appeared to play important role in the adaptation of the rice cultivar to DSR conditions) was validated by RT-qPCR following the MIQE guidelines. Total RNA was isolated in two biological replications using the TRIzol method (as described above) from 500 mg leaf and root tissues (collected in liquid nitrogen, that had previously been utilized for transcriptomics) of N-22 and IR64 grown by transplanting (control) and direct-sowing (treatment). RNAs of good quality/integrity were subjected to DNase I treatment (Qiagen, Germany) to remove any contaminating DNA. About 2.0 μg of total RNA was reverse-transcribed using Superscript III First-Strand Synthesis Kit (Invitrogen) in a final volume of 20 μl according to the manufacturer’s instructions. The synthesized cDNA was diluted ten times and used as a template for RT-qPCR validation of the selected genes (in two biological and three technical triplications) using KAPA SYBR fast qPCR Master mix 2 × (KAPA Biosystems) following the manufacturer’s instructions on a QIAquant 96 5plex machine (Qiagen, Germany). RT-qPCR was performed in 10 µl reaction mix containing 1.0 μl diluted cDNA, 0.1 μM each of the forward and reverse gene-specific primers, and 5.0 μl SYBR mix (KAPA Biosystems). Thermal cycler was programmed for an initial denaturation at 95 °C for 3 min, followed by 40 cycles each of 5 s denaturation at 94 °C, 20 s annealing at 60 °C and 40 s extension at 72 °C. Amplification data collection was set at the end of each extension step. The data was analyzed through melt curve analysis to check the specificity of PCR amplification. Details of the primers used for RT-qPCR validation are listed in Supplementary Table S1. The relative gene expression (fold change) was determined by the 2^−ΔΔCt^ method following the procedure described by Livak and Schmittgen^107^ using actin and tubulin as internal references.

### Validation based on morphophysiological/agronomic performance of plants

To validate the effects of the method of planting on the growth and development of rice, germination/seedling-vigor, number of tillers, and the number of productive tillers/panicles were assessed. To validate the effects on germination/seedling-vigor, 15 rice seeds were sown directly in the dry soil having soil moisture content ~ 50% lesser than that of the wet soil. Growth/development of plants and number of tillers per plant were also recorded at the age of 50 days for N-22, and at 60 days for IR64. The number of panicles per plant was recorded at the grain-filling stage. For this purpose, data were collected from a minimum of three pots for each cultivar grown by different planting methods.

To evaluate/validate the effects of different planting methods on the uptake, transport/mobilization, and accumulation of nutrients, total phosphorus (P) content in different (root, leaf, and panicle) tissues was determined using the Vanadate-molybdate photometric method^108^ with minor modifications. The plant tissue (collected after panicle emergence) was first dried at 60 °C until reached a constant weight. One gram of dried tissue sample was digested/extracted in 150 ml conical flak containing 10 ml of HNO_3_ and 3.5 ml of HClO_4_ by heating at 200 °C for 90 min using an acid-proof digestion chamber fitted with a fume-exhaust system. The extract was cooled down to room temperature, volume was made up to 25 ml with double distilled water (ddH_2_O), filtered with Whatman filter paper No. 42, and finally, the volume was made up to 100 ml with ddH_2_O. A 25 ml aliquot of the extract and 10 ml of Vanadate-molybdate solution were mixed in a 50 ml volumetric flask, and the volume was made up to 50 ml with ddH_2_O. The content was incubated for 10 min at room temperature for color development, and absorbance was recorded at 420 nm in triplicate. A standard curve was prepared for varying concentrations of P (0, 1, 1.5, 2, 2.5, 3, 3.5, 4.0, 4.5, and 5 mg) using orthophosphoric acid (H_3_PO_4_), color development, taking OD (as mentioned above), and plotting P concentration on X-axis while OD value at Y-axis. P content (%) in sample tissues was determined using the standard curve.

## Supplementary Information

Supplementary Figures.

Supplementary Table S1.

Supplementary Table S2.

Supplementary Table S3.

Supplementary Table S4.

Supplementary Table S5.

Supplementary Table S6.

Supplementary Table S7.

Supplementary Table S8.

Supplementary Table S9.

Supplementary Table S10.

Supplementary Table S11.

Supplementary Table S12.

Supplementary Table S13.

Supplementary Table S14.

Supplementary Table S15.

Supplementary Table S16.

Supplementary Table S17.

## Data Availability

The morphophysiological and biochemical data generated during this study are included in the Supplementary files. RNA-sequencing raw data are available at NCBI Sequence Read Archive (SRA) database (https://www.ncbi.nlm.nih.gov/sra) under the BioProject ID: PRJNA805549.
