# Author Correction: RNA-seq analysis reveals the genes/pathways responsible for genetic plasticity of rice to varying environmental conditions on direct-sowing and transplanting

**DOI:** 10.1038/s41598-022-07812-1

**Published:** 2022-03-02

**Authors:** Suresh Kumar, Karishma Seem, Santosh Kumar, Trilochan Mohapatra

**Affiliations:** 1 Division of Biochemistry, ICAR-Indian Agricultural Research Institute, New Delhi, 110012 India; 2 Decode Genomics Private Limited, New Delhi, India; 3 Indian Council of Agricultural Research, New Delhi, India

Correction to: *Scientific Reports* 10.1038/s41598-022-06009-w, published online 10 February 2022.

The original version of this Article contained a repeated error in the BioProject ID number where “PRJNA805549” was incorrectly given as “PRJNA 828053”.

The original Article has been corrected.

