# Supplementary material for: RNA-seq analysis reveals the genes/pathways responsible for genetic plasticity of rice to varying environmental conditions on direct-sowing and transplanting
Source: Sci Rep. 2022 Feb 10;12:2241. doi: 10.1038/s41598-022-06009-w (PMC8831524; doi:10.1038/s41598-022-06009-w)
Supplement: Supplementary file 1 — Supplementary Figures. [file 41598_2022_6009_MOESM1_ESM.docx]

**RNA-seq analysis reveals the genes/pathways responsible for genetic plasticity of rice to varying environmental conditions on direct-sowing and transplanting**

**Suresh Kumar, Karishma Seem, Santosh Kumar, and Trilochan Mohapatra**

**Supplementary Fig. S1.** Germination-vigor of rice cultivars (Nagina-22 and IR64) on dry/direct-sowing.


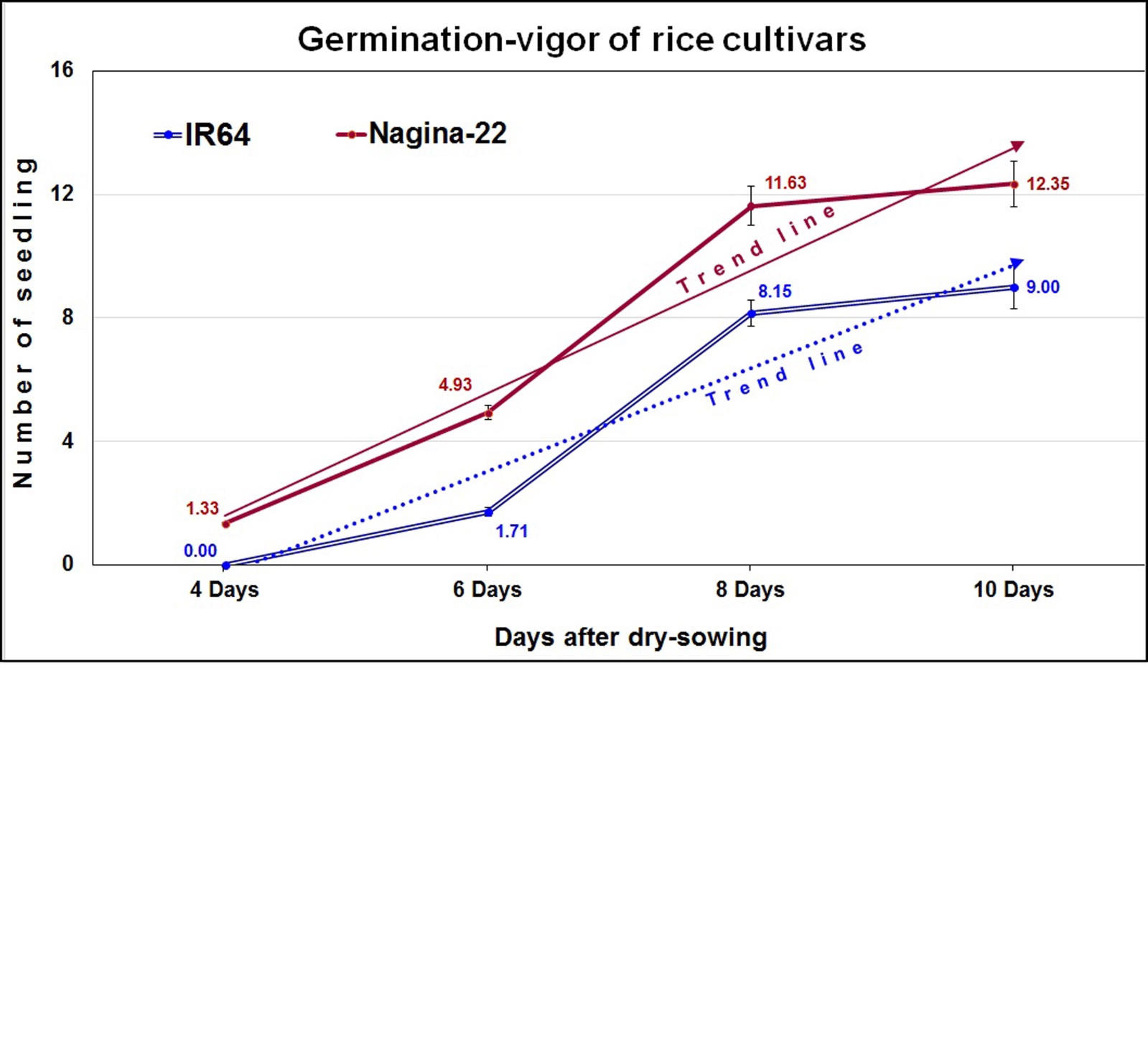


**Supplementary Fig. S2.** Seedling-vigor of rice cultivars (Nagina-22 and IR64) grown by dry/direct-sowing in pots.


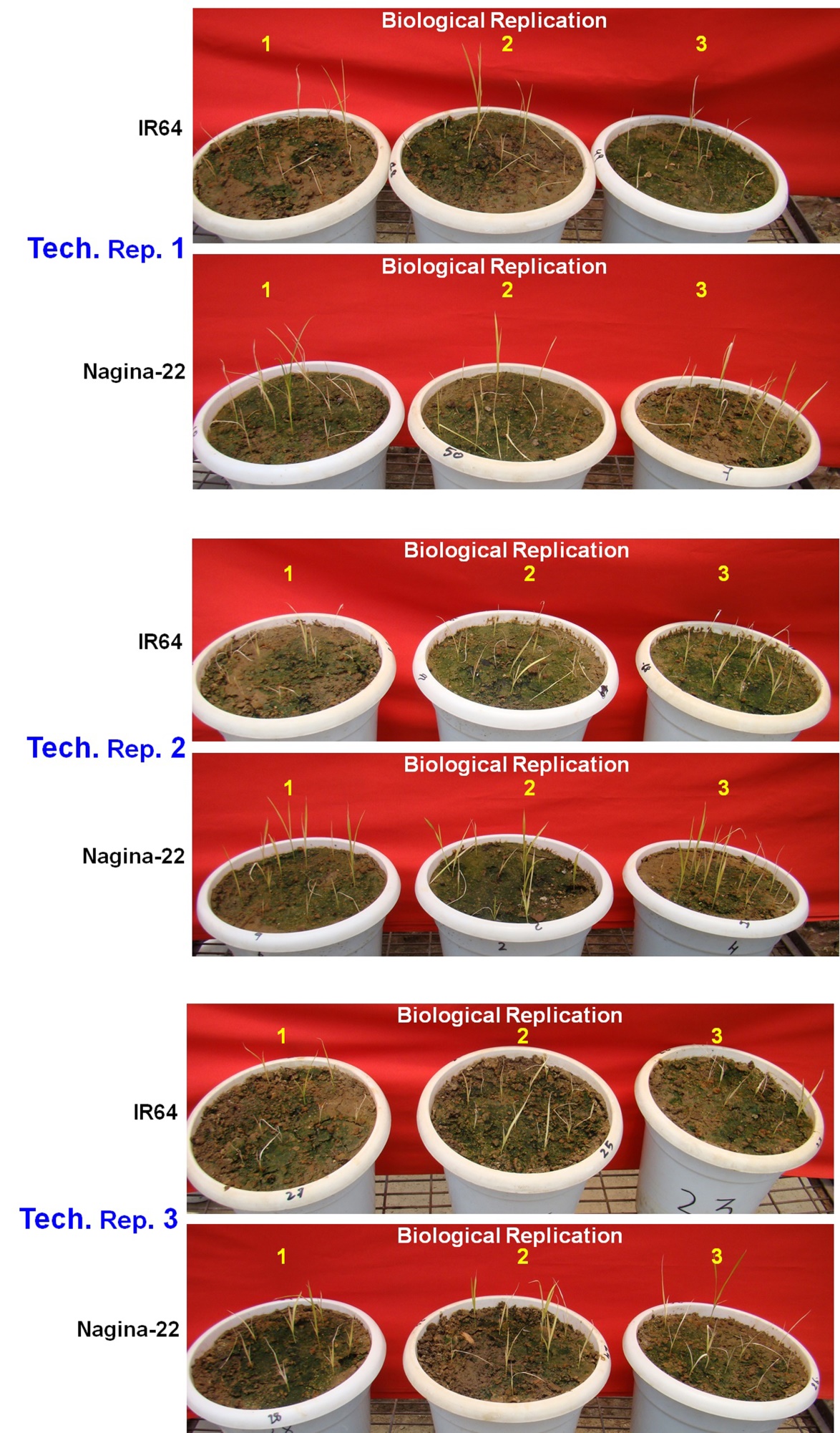


**Supplementary Fig. S3.** Seedling vigor of the rice cultivars (Nagina-22 and IR64) grown by nursery raising.


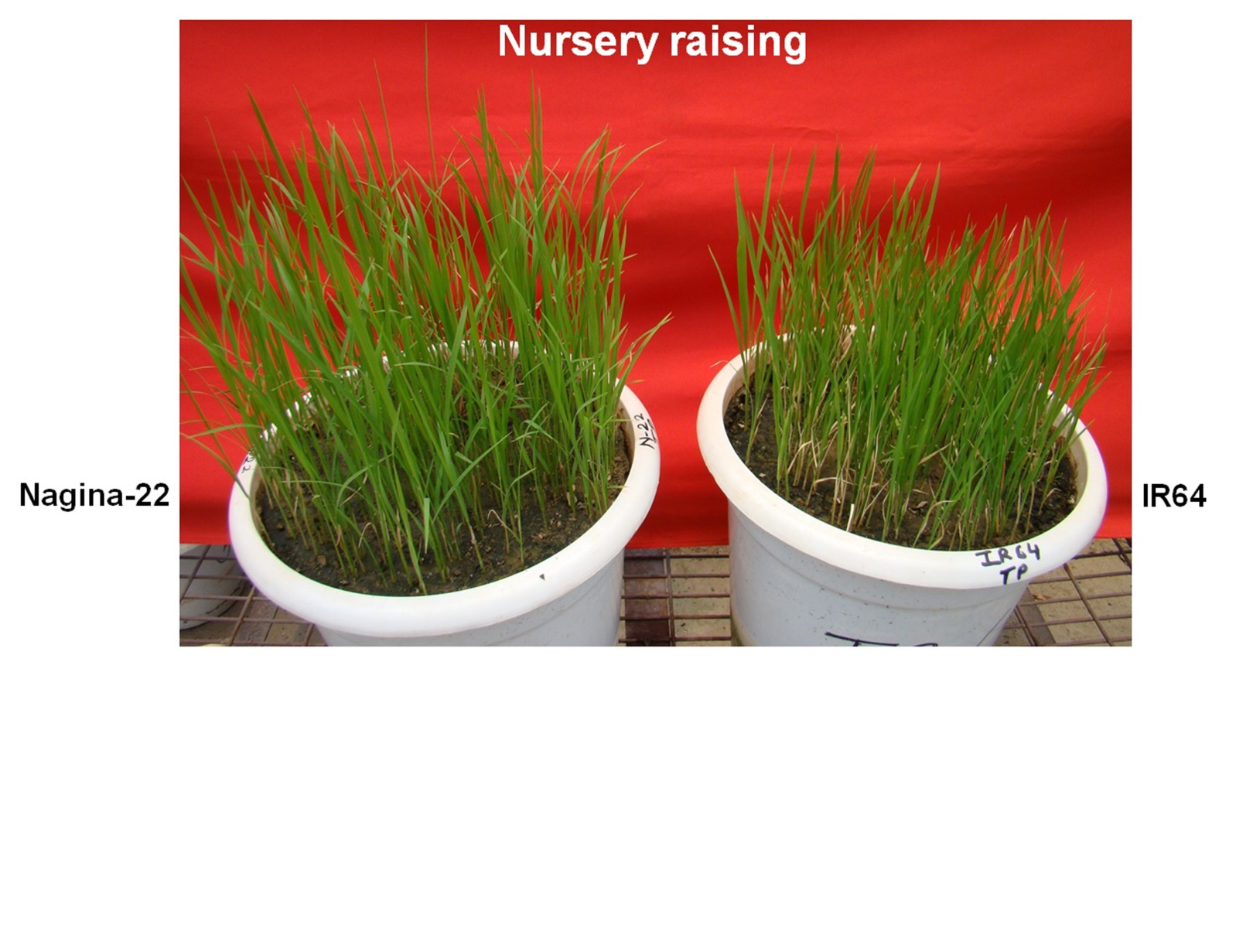


**To be used for transplanting**

**Supplementary Fig. S4.** Variation in characteristic features of root of rice cultivars (IR64 and Nagina-22) at the age of 30 days grown under direct-sown conditions. (**A**) Total root length, (**B**) number of root tips, (**C**) root surface area, and (**D**) root volume.


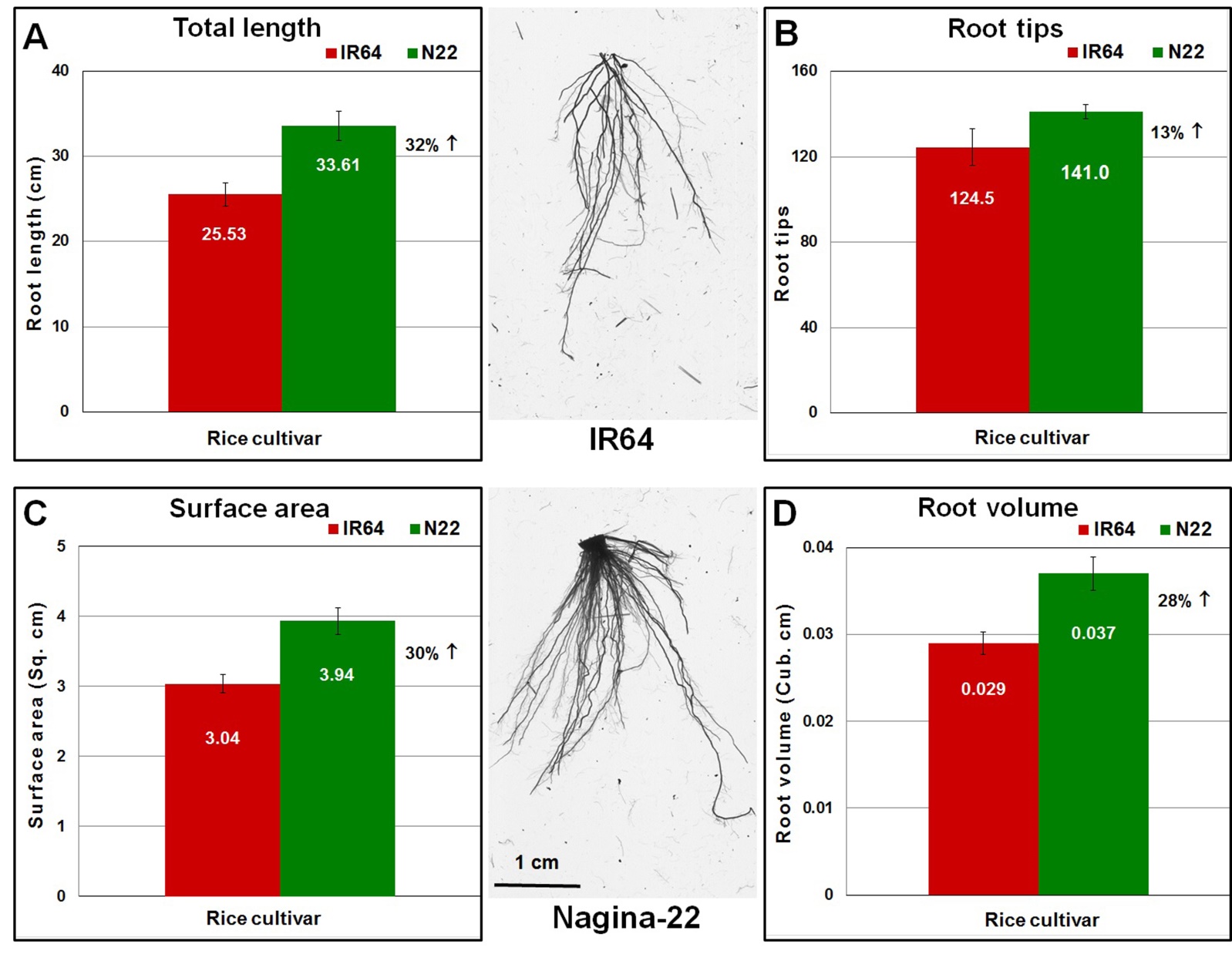


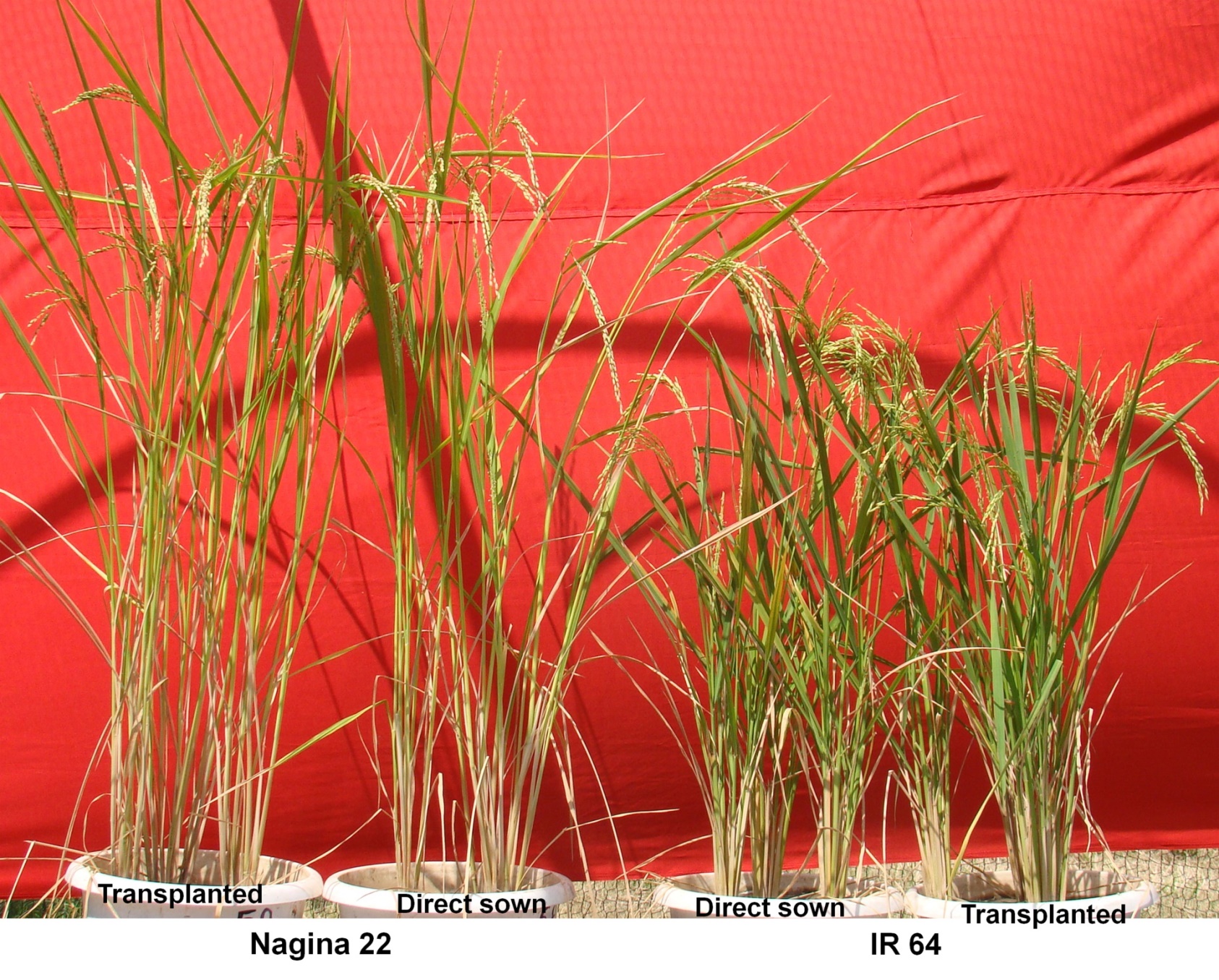
**Supplementary Fig. S5.** Performance (agronomic) of the rice cultivars (Nagina-22 and IR64) under transplanted and direct sown conditions.

**Nagina-22 IR64**

**Supplementary Fig. S6.** Total phosphorus content in different tissues of rice cultivars grown by transplanting and direct sowing. (**A**) Root, (**B**) leaf, and (**C**) panicle. For estimation of P content, leaf and root tissues were collected at the panicle initiation stage, and finally the P content was estimated in the mature panicle/seeds.


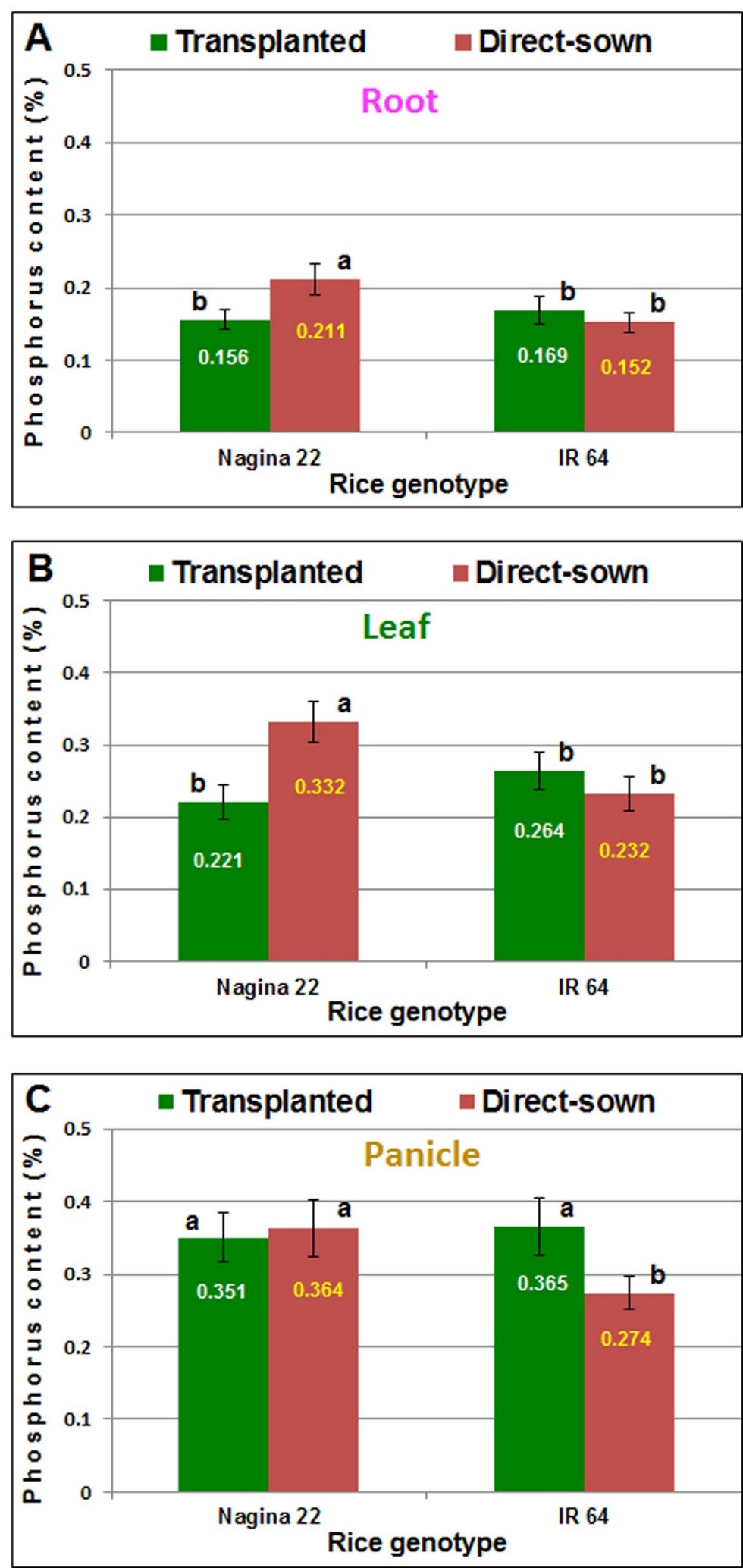


**Supplementary Fig. S7.** Cultivar-specific differential expression of genes on direct-sowing. (**A**) IR64, and (**B**) Nagina-22. Change in expression (Up- up-regulation, or Dn- down-regulation) was calculated based on the expression level of the genes on direct sowing (treatment) over transplanting (control).


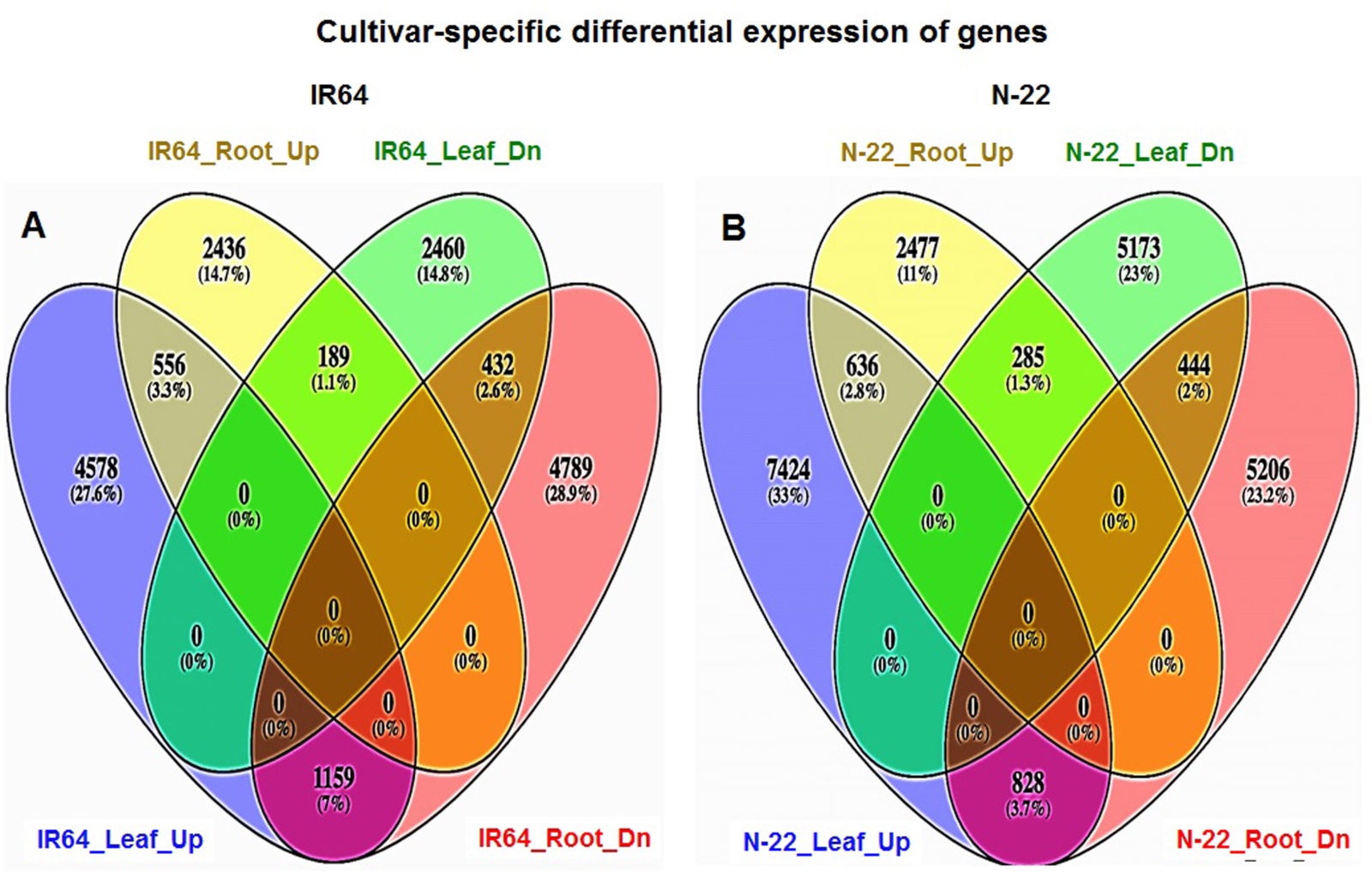


**Supplementary Fig. S8.** Gene ontology (GO) analysis of under-represented biological process on direct-sowing in leaf of rice cultivars. (**A**) Under-represented GO terms in the leaves of Nagina-22, and (**B**) under-represented GO terms in the leaf of IR64.


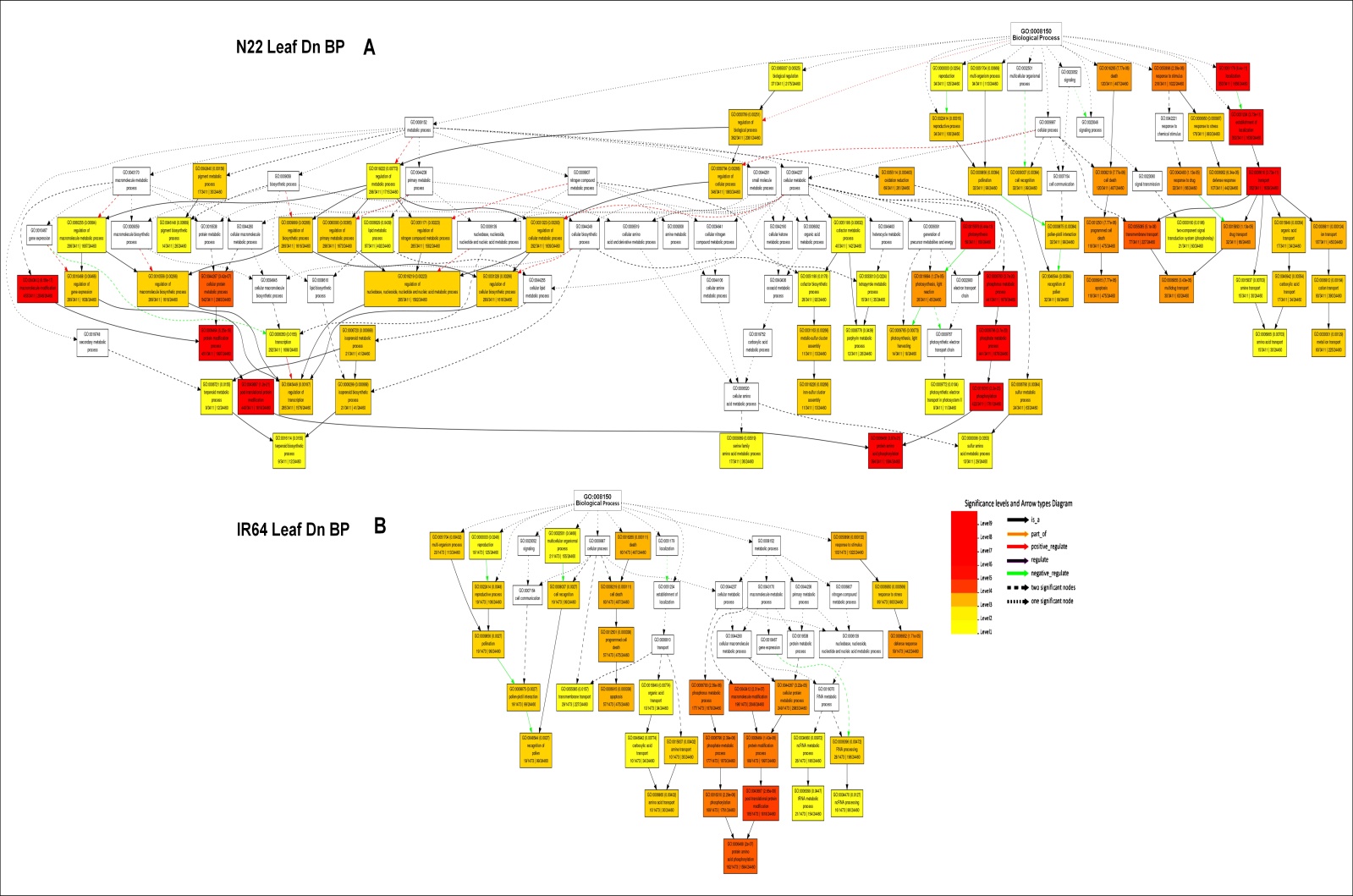


**Supplementary Fig. S9.** Gene ontology (GO) analysis of under-represented molecular function on direct-sowing in leaf of rice cultivars. (**A**) Under-represented GO terms in the leaf of Nagina-22, and (**B**) under-represented GO terms in the leaf of IR64.


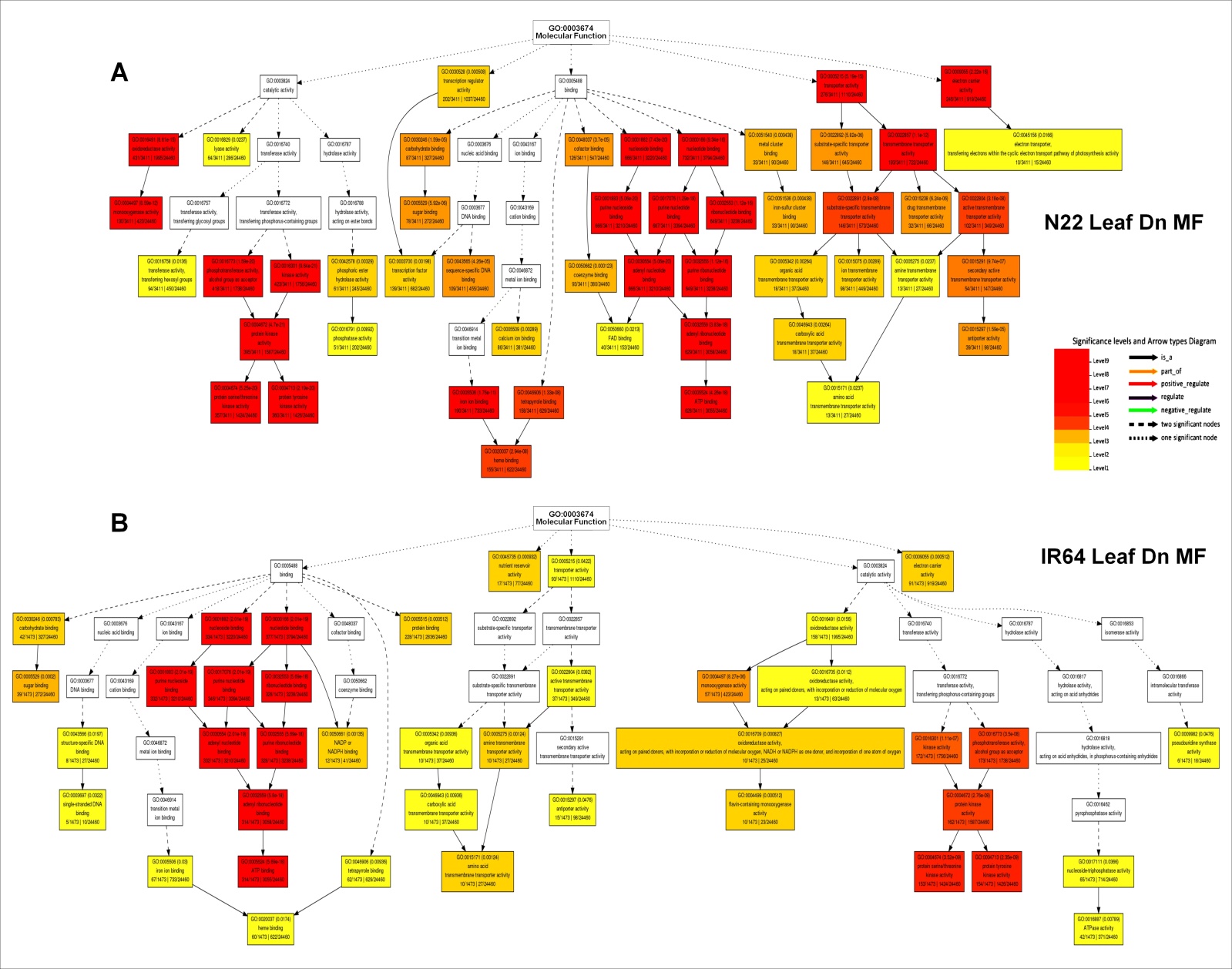


**Supplementary Fig. S10.** Gene ontology (GO) analysis of enriched cellular component on direct-sowing in leaf of rice cultivars. (**A**) Over-represented GO terms in the leaf of Nagina-22, and (**B**) over-represented GO terms in the leaf of IR 64 rice.


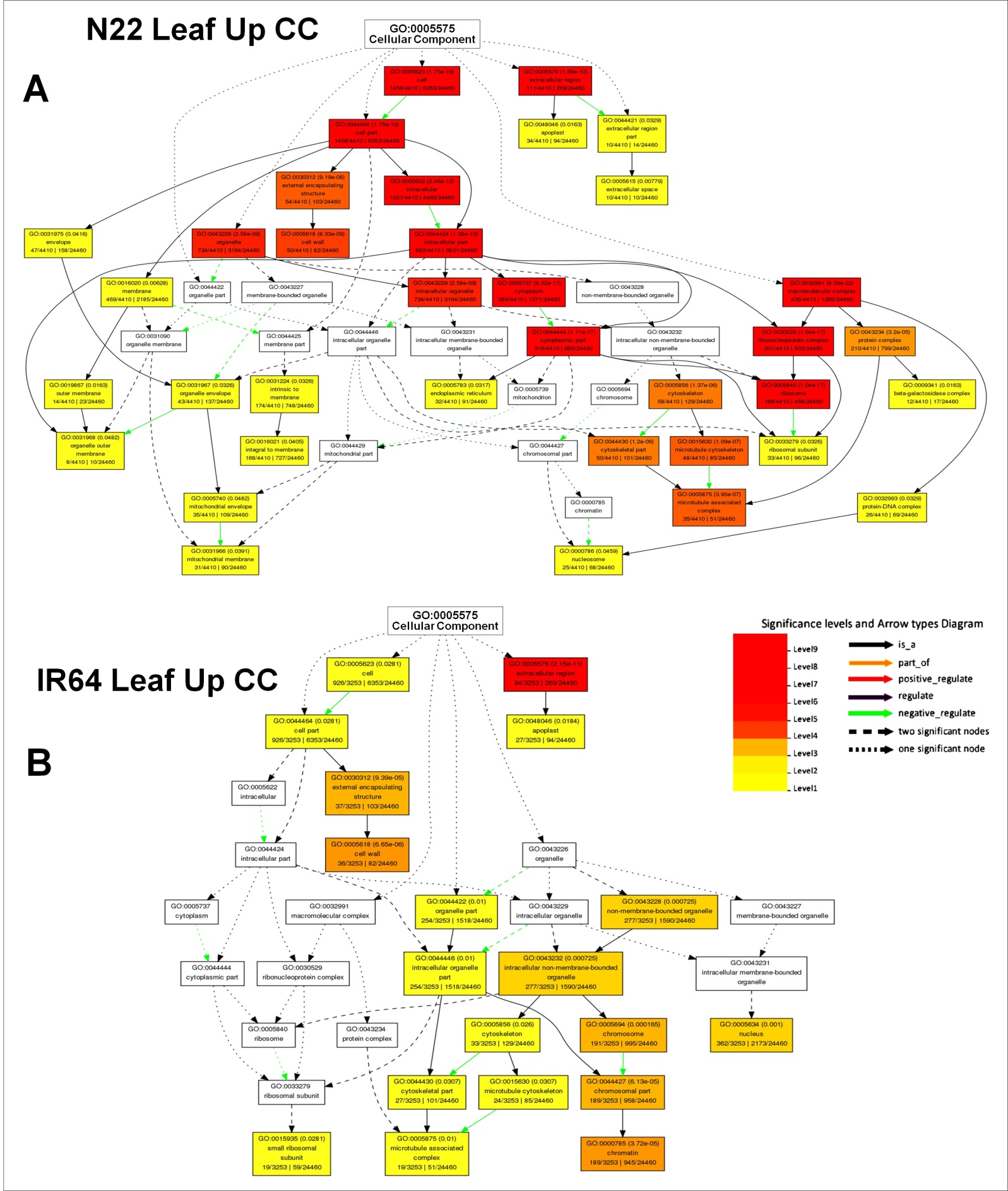


**Supplementary Fig. S11.** Gene ontology (GO) analysis of under-represented cellular components on direct-sowing in leaf of rice cultivars. (**A**) Under-represented GO terms in the leaf of Nagina-22, and (**B**) under-represented GO terms in the leaf of IR64.


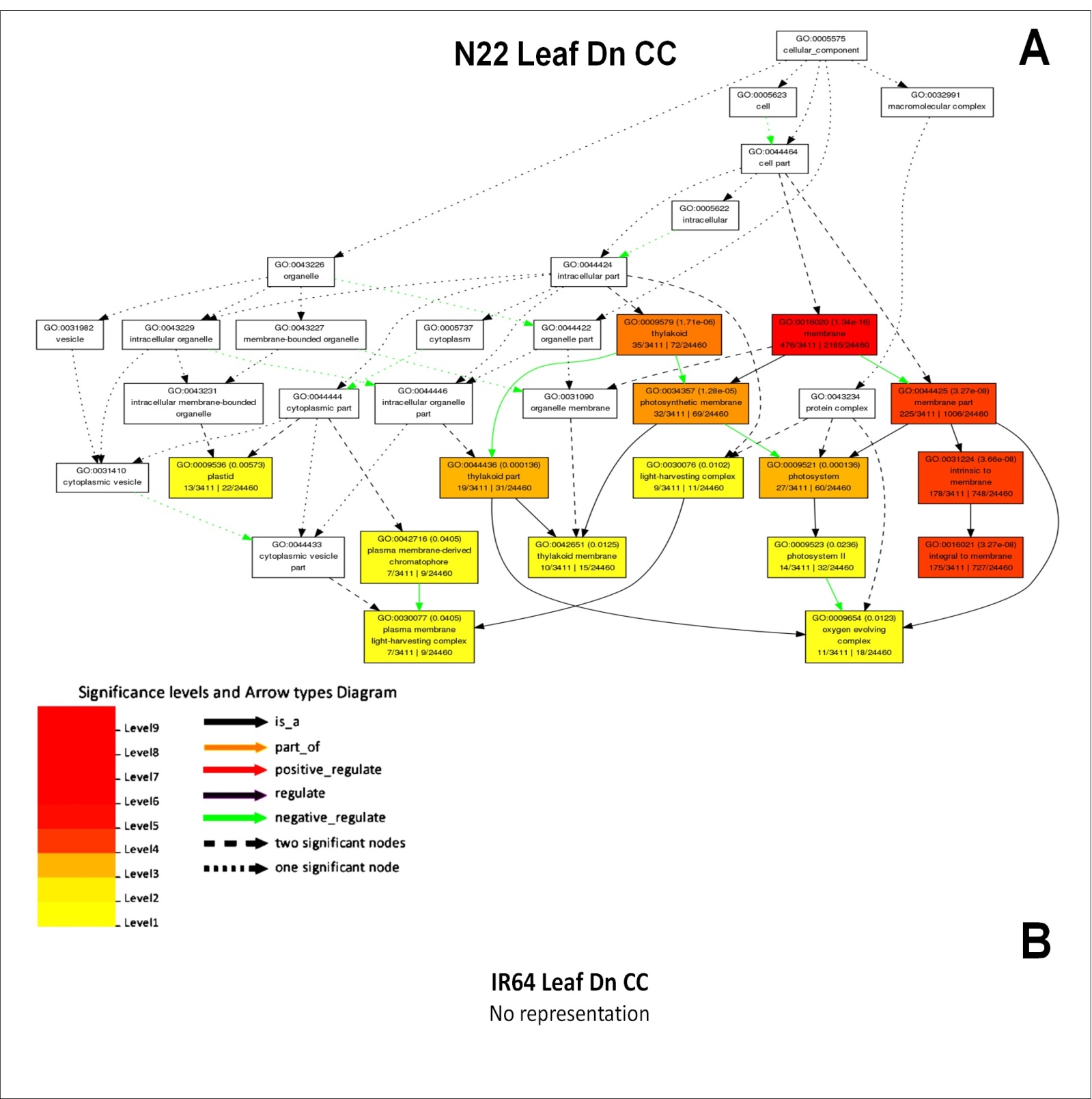


**Supplementary Fig. S12.** Gene ontology (GO) analysis of over-represented biological process on transplanting over direct-sowing in leaf of contrasting rice cultivars. (**A**) Over-represented GO terms in the leaf of Nagina-22, and (**B**) over-represented GO terms in the leaf of IR64.


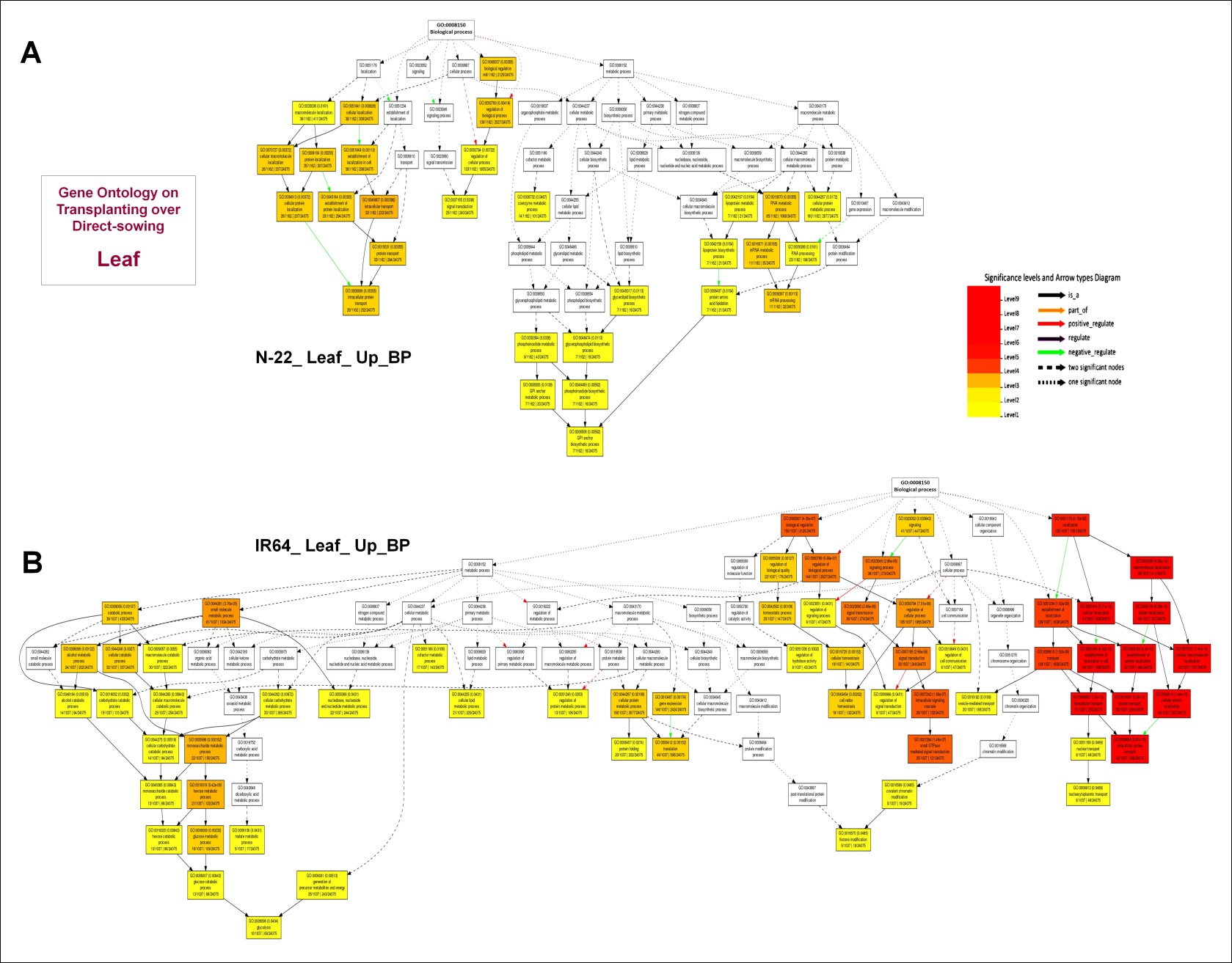


**Supplementary Fig. S13.** Gene ontology (GO) analysis of under-represented biological process on transplanting over direct-sowing in leaf of contrasting rice cultivars. (**A**) Under-represented GO terms in the leaf of Nagina-22, and (**B**) under-represented GO terms in the leaf of IR64.


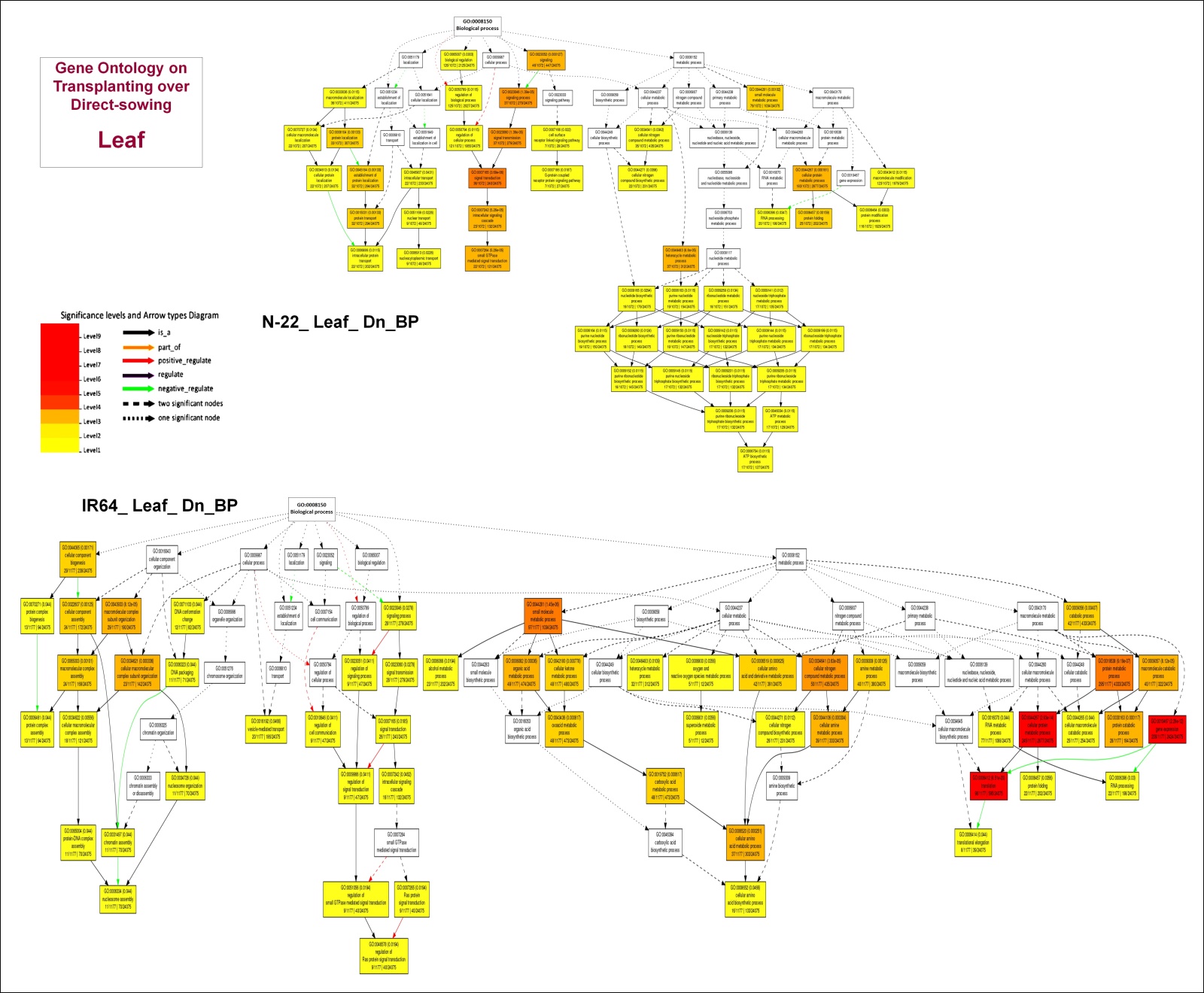


**Supplementary Fig. S14.** Gene ontology (GO) analysis of over-represented biological process on transplanting over direct-sowing in root of contrasting rice cultivars. (**A**) Over-represented GO terms in the roots of Nagina-22, and (**B**) over-represented GO terms in the roots of IR64.


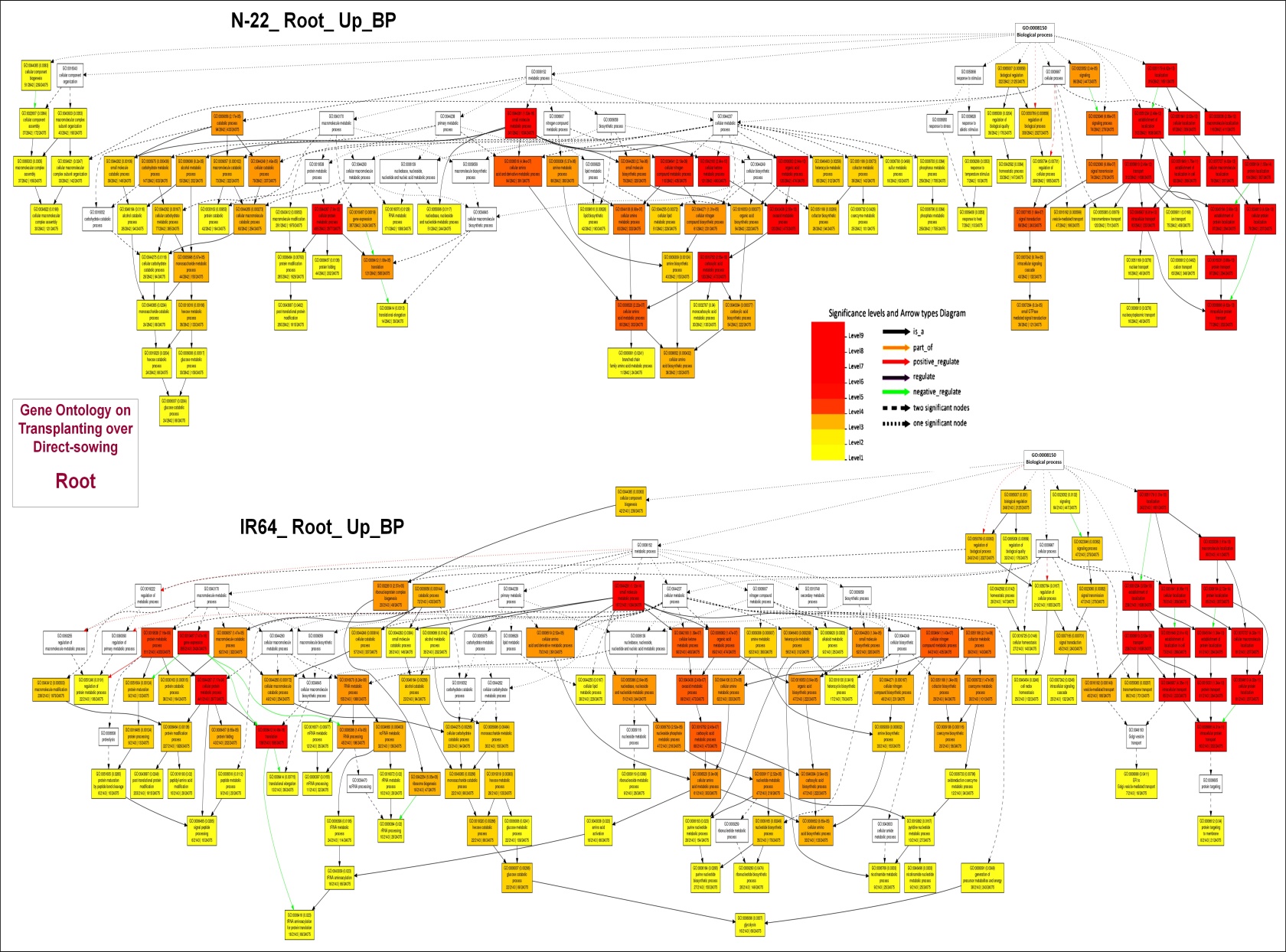


**Supplementary Fig. S15.** Gene ontology (GO) analysis of under-represented biological process on transplanting over direct-sowing in root of contrasting rice cultivars. (**A**) Under-represented GO terms in the roots of Nagina-22, and (**B**) under-represented GO terms in the roots of IR64.

**
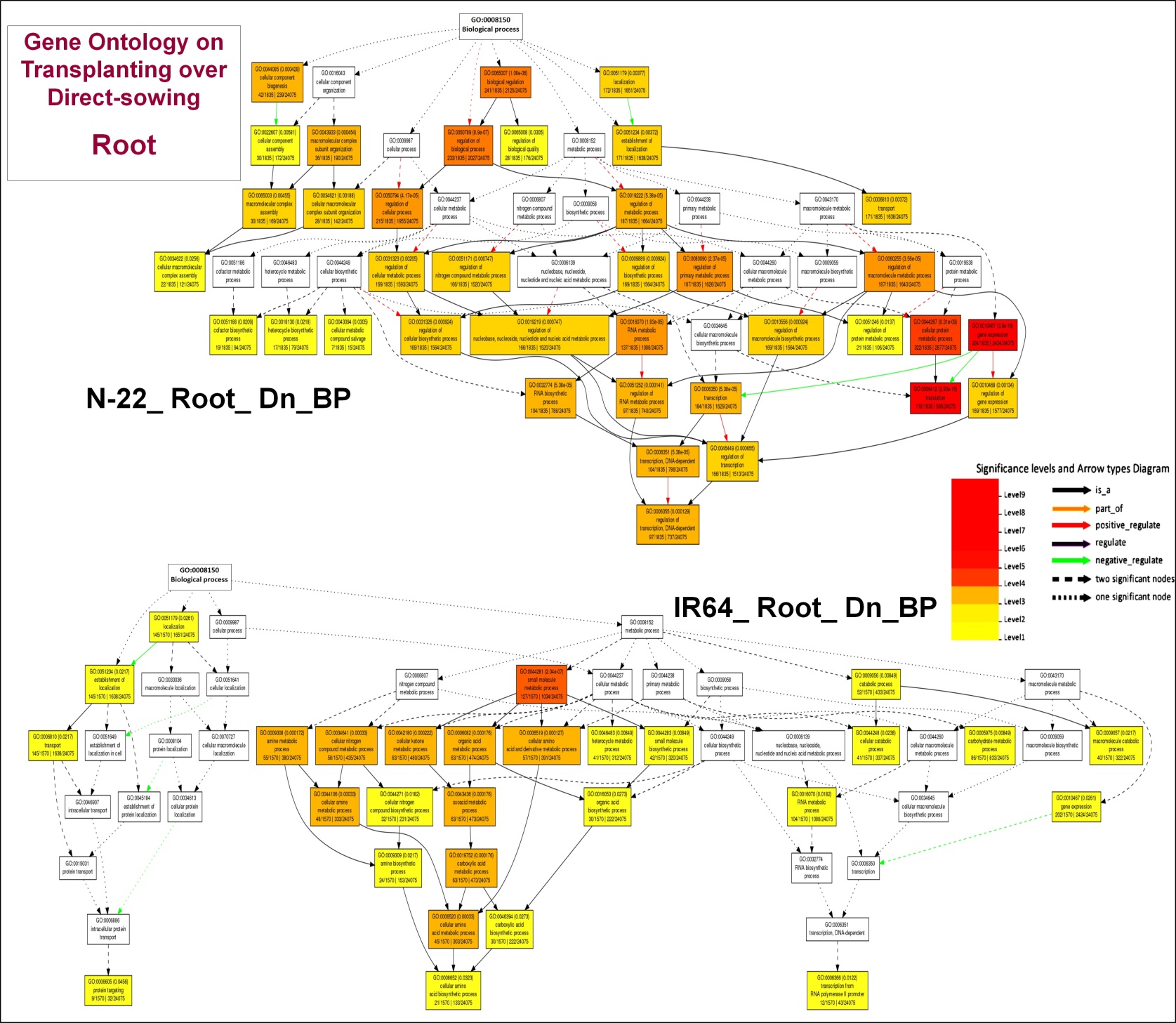
**

**Supplementary Fig. S16.** Heat map showing differential expression of some of the genes associated with nutrient reservoir activities in the contrasting rice cultivars in the leaves and roots (**A**) under direct-sown over transplanted conditions (control) and (**B**) on transplanting over direct-sowing (control).

**
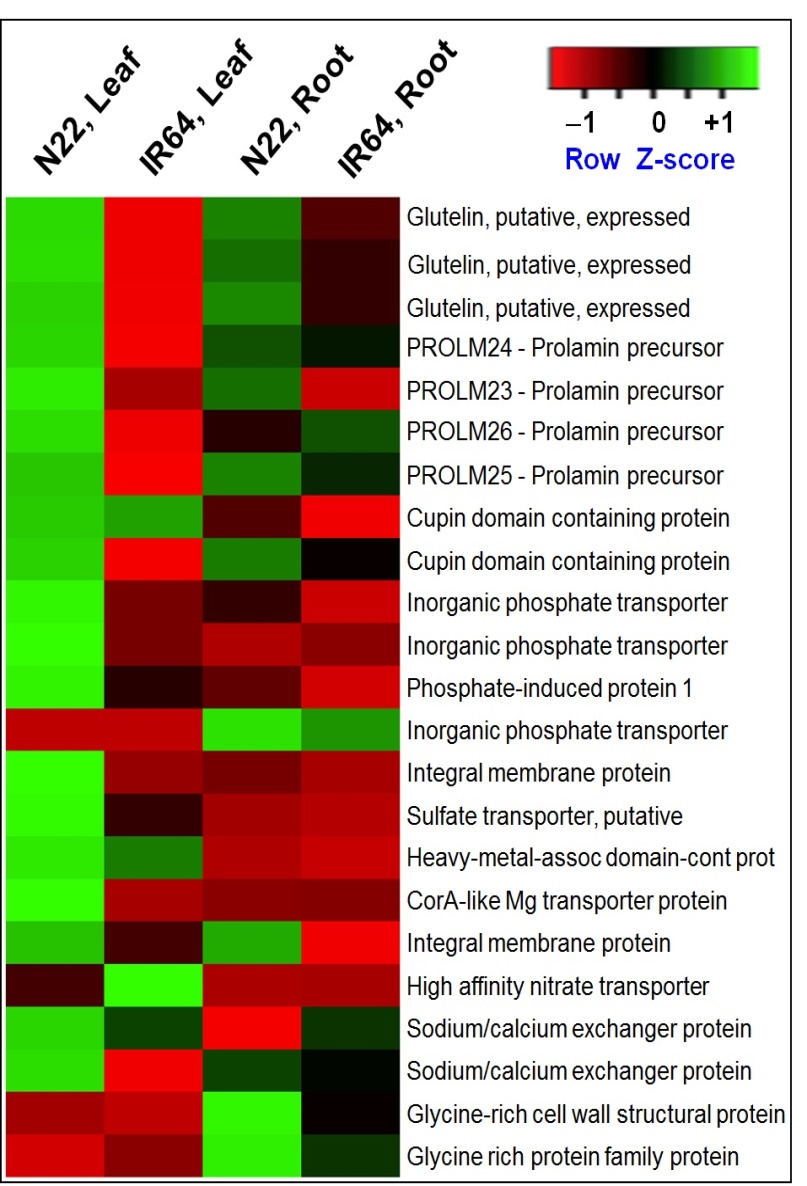
**

**A Direct-sown over transplanted**

**B Transplanted over direct-sown**

**A Direct-sown over transplanted**


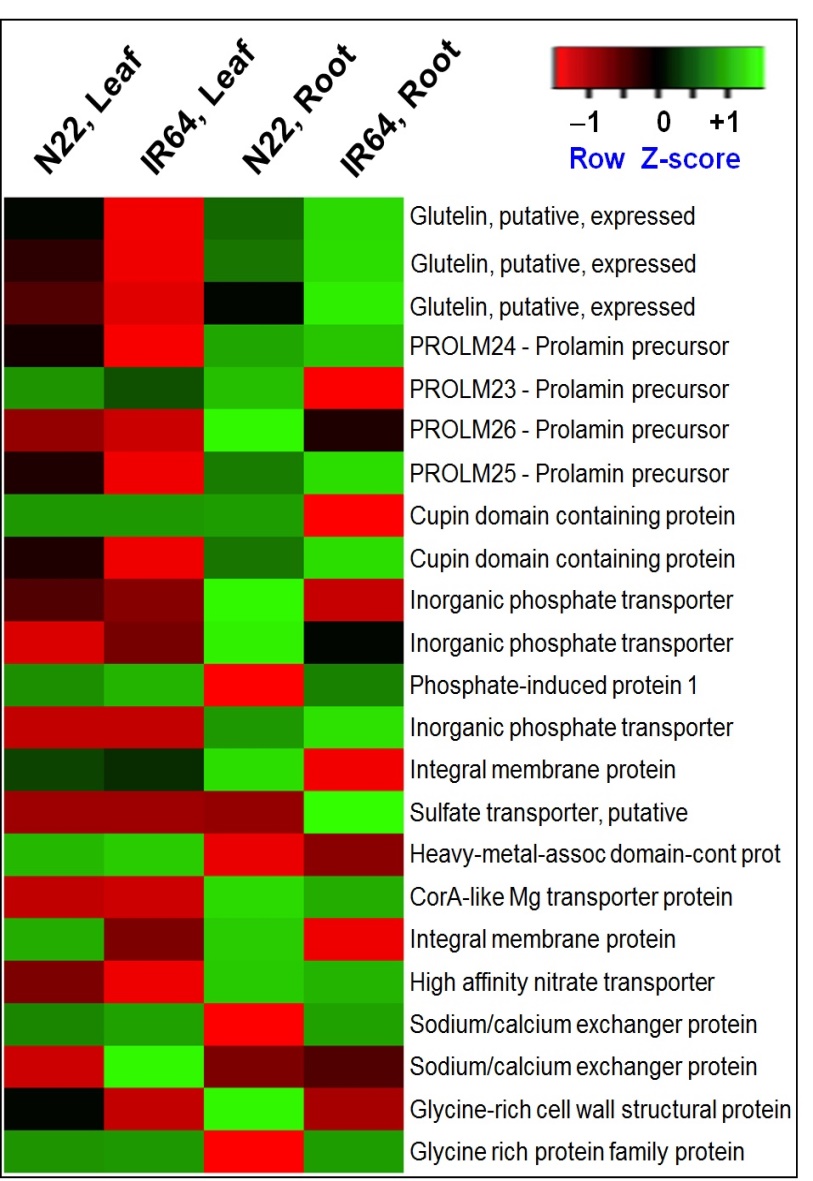


**Supplementary Fig. S17.** Heat map showing differential expression of genes for transcription factors in the contrasting rice cultivars in the leaves and roots (**A**) under direct-sown over transplanted (control) conditions and (**B**) on transplanting over direct-sowing (control).

**Supplementary Fig. S17.** Heat map showing differential expression of genes for transcription factors in the contrasting rice cultivars in the leaves and roots (**A**) under direct-sown over transplanted (control) conditions and (**B**) on transplanting over direct-sowing (control).

**B Transplanted over direct-sown**

**A Direct-sown over transplanted**


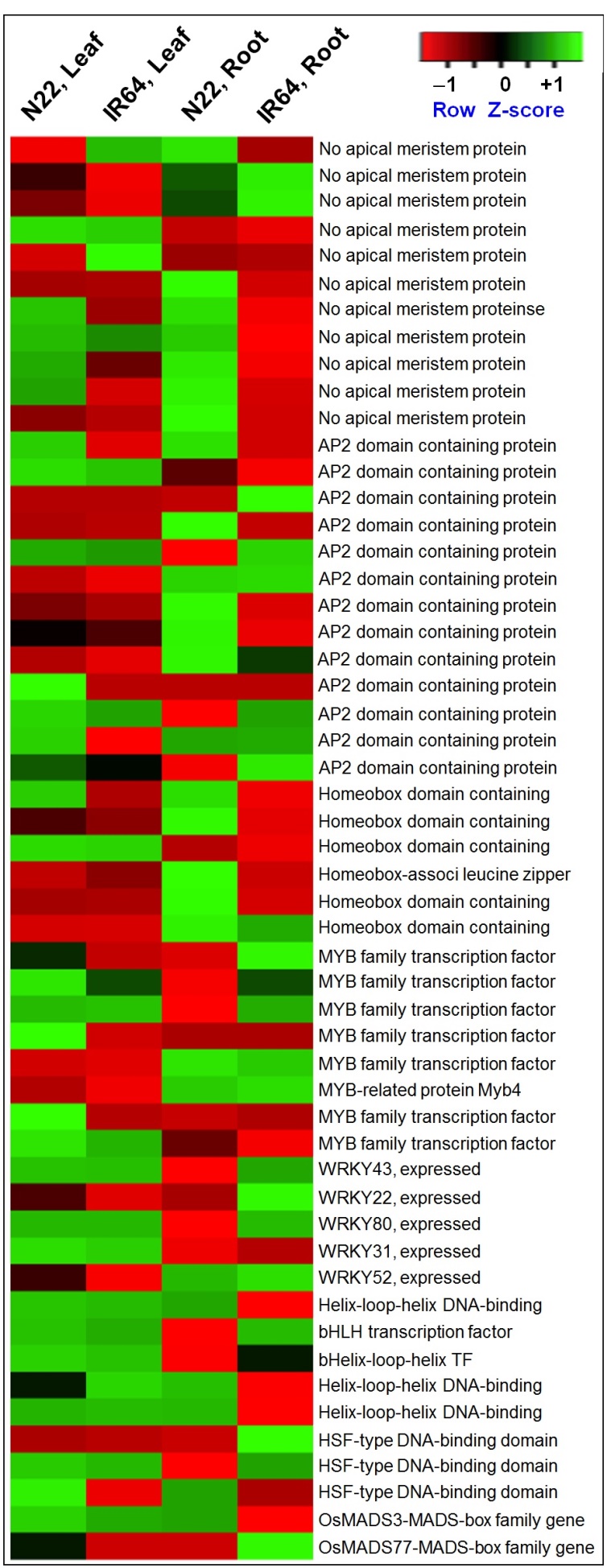
**
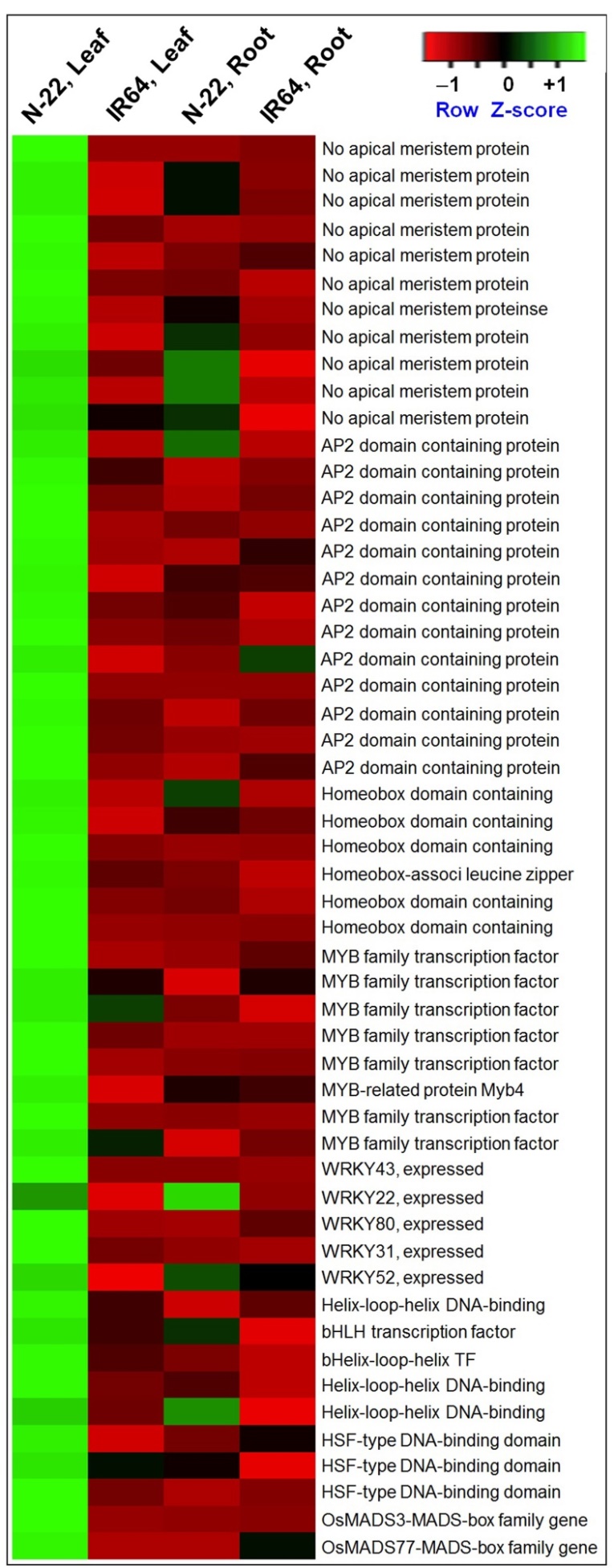
**

**Supplementary Fig. S18.** Heat map showing differential expression of some of the important genes associated with different pathways under direct-sown conditions in leaf and root of rice cultivars.


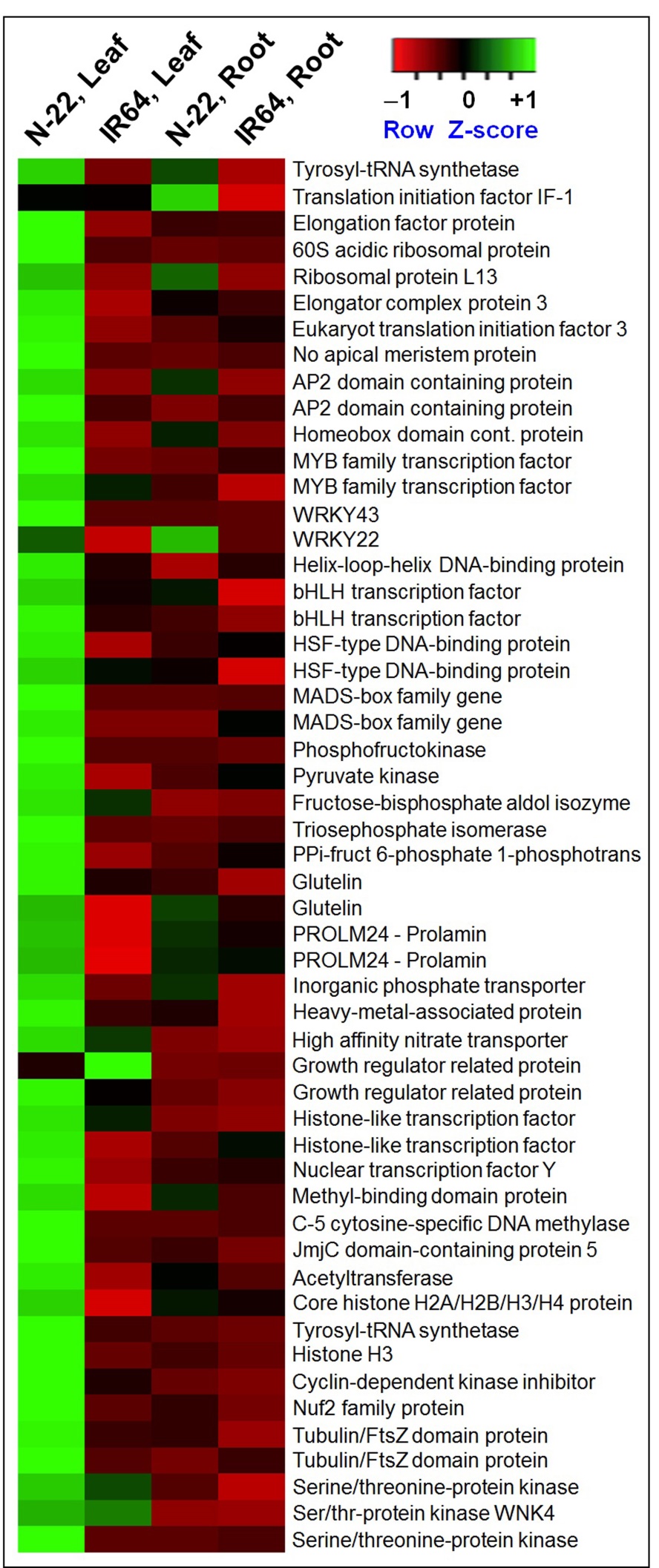


**
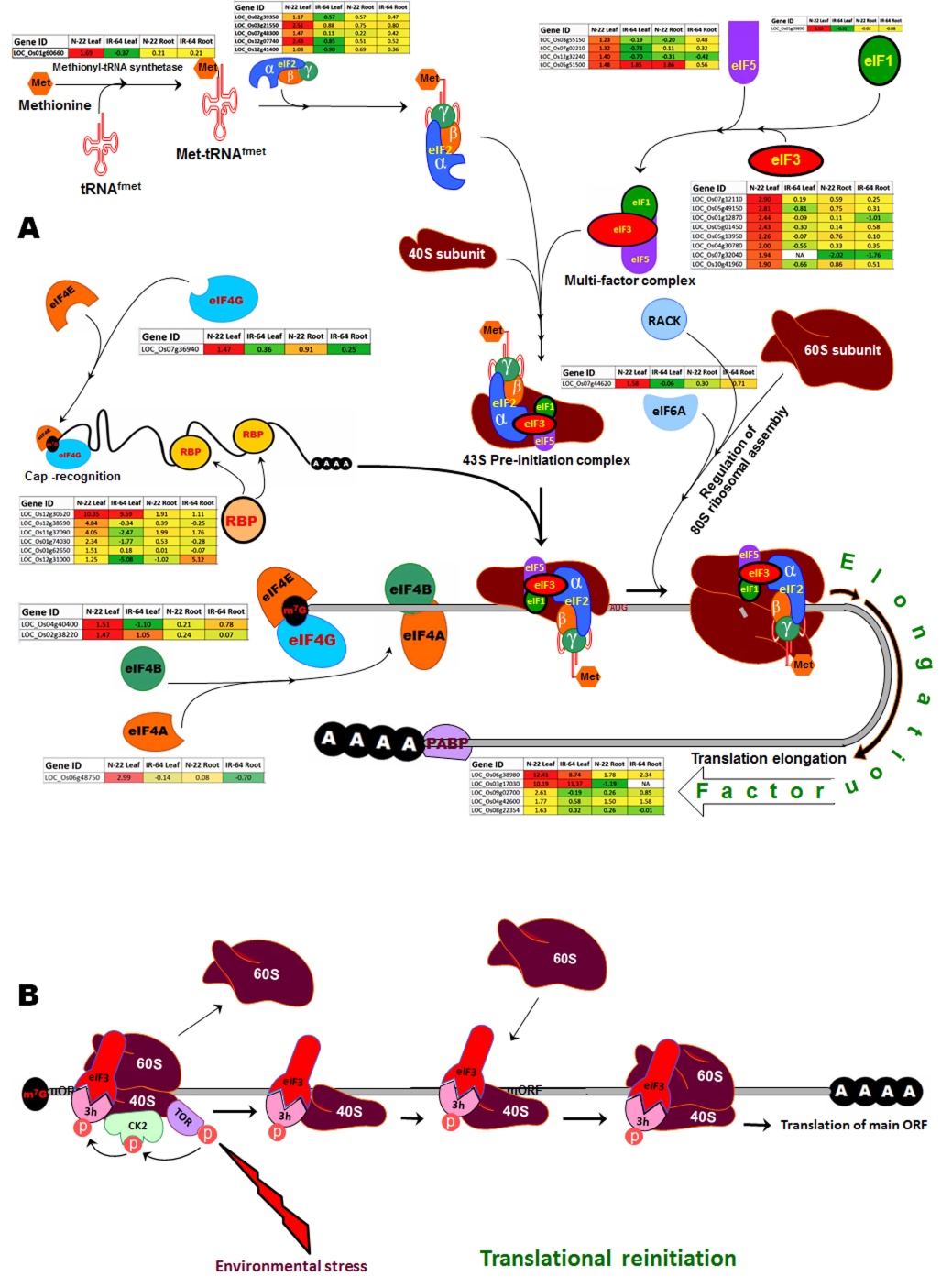
Supplementary Fig. S19.** A proposed model for the regulation of translational machinery in Nagina 22 rice cultivar under direct-sown [over transplanted (control)] conditions. (**A**) Initiation of translation is controlled by cap-recognising initiation factors (eIF4E and eIF4G), and RNA-binding protein (RBP) protects the mRNA. 43S pre-initiation complex (PIC) is formed by the association of 40S ribosomal subunit, a charged methionyl-tRNA^fmet^, eIF2, eIF1, eIF3 and eIF5. The 43S PIC associates with the eIF4 complex and binds to mRNA. The ATP-dependent helicase activity of eIF4A unwinds mRNA secondary structures and eIF6 along with RACK proteins regulate 80S ribosomal assembly which continues to translation of mRNA. (**B**) Regulation of translational reinitiation in N-22 during environmental stresses under direct-sown conditions. Environmental stress triggers phosphorylation of specific eIF, which has drastic effects on the rate of translation either by repressing or up-regulating translation. During environmental stress, CK2 (casein kinase 2) phosphorylates eIF3 in association with TOR. After translation of upstream open reading frame (uORF), phosphorylation of eIF3h plays important role in reinitiation of translation. After disassembly of the ribosome subunits at uORF, the phosphorylated eIF3h allows 40S to recruit a new 60S at the AUG of main open reading frame (mORF) and reinitiate translation to meet the cellular demand (adopted from Merchante *et al*.^63^ and Galland *et al*.[^7^](https://www.biorxiv.org/content/10.1101/660860v1.full#ref-37)^3^).
