# Supplementary material for: RNA-seq analysis reveals the genes/pathways responsible for genetic plasticity of rice to varying environmental conditions on direct-sowing and transplanting
Source: Sci Rep. 2022 Feb 10;12:2241. doi: 10.1038/s41598-022-06009-w (PMC8831524; doi:10.1038/s41598-022-06009-w)
Supplement: Supplementary file 2 — Supplementary Table S1. [file 41598_2022_6009_MOESM2_ESM.pdf]

RNA-seq analysis reveals the genes/pathways responsible for genetic plasticity of rice to varying environmental conditions on direct-sowing and transplanting

Suresh Kumar, Karishma Seem, Santosh Kumar, and Trilochan Mohapatra

Supplementary Table S1. List of the primers used for RT-qPCR validation of the randomly selected differentially expressed genes (DEGs) in leaf and root of the rice cultivars (Nagina-22 and IR64).

**Gene**	**Gene LOC**	**Forward Primer (5′→ 3′)**	**Reverse Primer (5′→ 3′)**
OsGRF02	LOC_Os02g47280	GCGTACGGC GAGTCCAAGTA	GGGCGGCATTTCCACAG
OsGRF11	LOC_Os11g35030	GGAAGAGTGTTACTGGTGCATCT	AGTCCAAGCAGAGTGTTGCC
OsNF-YA8	LOC_Os10g25850	CATAGAACATCCCCTT	GAGATCTCCTGTAAAACC
OsNF-YB9	LOC_Os06g17480	GTTCCGCTACGTGCAGGTCCAT	ACGACATGGCCACGGGGTAA
OsNF-YC11	LOC_Os10g11580	ATGCTATTGTTGGTGAAAAC	GAAGATCTTTGTCAGGAAAA
Actin	LOC_Os03g50885	TTGCTGACAGGATGAGCAAG	TGGAATGTGCTGAGAGATGC
β-tubulin	LOC_Os01g59150	GCTGACCACACCTAGCTTTGG	AGGGAACCTTAGGCAGCATGT
